# MMP12 knockout prevents weight and muscle loss in tumor-bearing mice

**DOI:** 10.1186/s12885-021-09004-y

**Published:** 2021-12-04

**Authors:** Lingbi Jiang, Mingming Yang, Shihui He, Zhengyang Li, Haobin Li, Ting Niu, Dehuan Xie, Yan Mei, Xiaodong He, Lili Wei, Pinzhu Huang, Mingzhe Huang, Rongxin Zhang, Lijing Wang, Jiangchao Li

**Affiliations:** 1 Institute of Basic Medical Sciences, School of Life Sciences and Biopharmaceuticals, Guangdong Pharmaceutical University, No. 280 Waihuan Rd. E, Higher Education Mega Center, Guangzhou, 510006 China; 2 The State Key Laboratory of Oncology in South China, Sun Yat-sen University Cancer Center, Guangzhou, 510060 China; 3 The Sixth Affiliated Hospital, Sun Yat-sen University, Guangzhou, 510060 China; 4 Guangdong Province Key Laboratory for Biotechnology Drug Candidates, Guangdong Pharmaceutical University, Guangzhou, 510006 China

**Keywords:** MMP12, Apc^Min/+^, Macrophage, IL-6, Cancer cachexia, Colorectal cancer

## Abstract

**Background:**

Colorectal cancer is a malignant gastrointestinal cancer, in which some advanced patients would develop cancer cachexia (CAC). CAC is defined as a multi-factorial syndrome characterized by weight loss and muscle loss (with or without fat mass), leading to progressive dysfunction, thereby increasing morbidity and mortality. Apc^Min/+^ mice develop spontaneous intestinal adenoma, which provides an established model of colorectal cancer for CAC study. Upon studying the Apc^Min/+^ mouse model, we observed a marked decrease in weight gain beginning around week 15. Such a reduction in weight gain was rescued when Apc^Min/+^ mice were crossed with MMP12^−/−^ mice, indicating that MMP12 has a role in age-related Apc^Min/+^-associated weight loss. As a control, the weight of MMP12^−/−^ mice on a weekly basis, their weight were not significantly different from those of WT mice.

**Methods:**

Apc^Min/+^; MMP12^−/−^ mice were obtained by crossing Apc^Min/+^ mice with MMP12 knockout (MMP12 ^−/−^) mice. Histological scores were assessed using hematoxylin-eosin (H&E) staining. MMP12 expression was confirmed by immunohistochemistry and immunofluorescence staining. ELISA, protein microarrays and quantitative Polymerase Chain Reaction (qPCR) were used to investigate whether tumor could up-regulate IL-6. Cell-based assays and western blot were used to verify the regulatory relationship between IL-6 and MMP12. Fluorescence intensity was measured to determine whether MMP12 is associated with insulin and insulin-like growth factor 1 (IGF-1) in vitro. MMP12 inhibitors were used to explore whether MMP12 could affect the body weight of Apc^Min/+^ mice.

**Results:**

MMP12 knockout led to weight gain and expansion of muscle fiber cross-sectional area (all mice had C57BL/6 background) in Apc^Min/+^ mice, while inhibiting MMP12 could suppress weight loss in Apc^Min/+^ mice. MMP12 was up-regulated in muscle tissues and peritoneal macrophages of Apc^Min/+^ mice. IL-6 in tumor cells and colorectal cancer patients is up-regulation. IL-6 stimulated MMP12 secretion of macrophage.

**Conclusions:**

MMP12 is essential for controlling body weight of Apc ^Min/+^ mice. Our study shows that it exists the crosstalk between cancer cells and macrophages in muscle tissues that tumor cells secrete IL-6 inducing macrophages to up-regulate MMP12. This study may provide a new perspective of MMP12 in the treatment for weight loss induced by CAC.

**Supplementary Information:**

The online version contains supplementary material available at 10.1186/s12885-021-09004-y.

## Background

Colorectal cancer (CRC) is the third most common malignant tumor in the worldwide in 2020, with poor prognosis and low survival rate, which seriously affects life quality of patients [1–4]. With CRC, 50-61% of patients developed cancer cachexia (CAC). However, CAC remains clinically underemphasized, and its underlying mechanism is not yet fully understood. Previous studies suggest that CAC is, to some extent, related to the local and systemic tumor immune responses and metabolic disorders [5].

Clinically, ongoing loss of body weight and skeletal muscle mass is a late outcome of nearly 80% of patients with different types of cancer, including pancreatic cancer, esophageal cancer, gastric cancer, lung cancer, liver cancer and CRC, and the mortality rate is as high as 30% [6–8]. More attention has been paid to CAC in recent years, and great progress has been made in the diagnosis and treatment of CRC [1, 9]. A standard treatment of CRC is a combination of surgery, chemotherapy and radiotherapy, but it fails to significantly reduce mortality rate, which is closely related to CAC syndrome in patients with advanced CRC [10].

Cancer cachexia is mainly characterized by weight loss. This syndrome is multifactorial, there are a complex interaction of tumor and host factors. Weight loss in patients include the loss of both adipose tissue and skeletal muscle mass, weight loss is mainly from the fat and the muscle. The Apc^Min/+^ mouse is accepted as an established model of colorectal cancer and cachexia [11, 12]. This mouse model with cachexia is the gradual progression of tumor development and muscle wasting that is more physiologically related to human disease, compared to tumor implant models.

MMP12, a matrix metalloprotease, also known as macrophage metalloelastase, belongs to the endoproteolytic enzyme family. Its activity is dependent on metal ions such as calcium and zinc, and it can degrade extracellular matrix. MMP12 was discovered in the morphological development of tadpoles. It is mainly derived from macrophages and is required for recruitment of monocytes [13–16]. MMP12 affects adipose tissue dilatation, the number of macrophages, body weight and body fat in MMP12-knockout mice fed with high fat diet [13]. Previous studies reported that MMP12 could specifically degrade insulin [17]. Researchers at the University of Washington confirmed that MMP12 regulates insulin sensitivity and is positively correlated with insulin resistance [18]. MMP12 was further identified as a therapeutic target for insulin-related metabolic diseases [13, 18]. Insulin plays an important role in disorders of glucose and lipid metabolism (e.g. impaired glucose tolerance and insulin resistance) are associated with body weight loss induced by CAC [19].

Some inflammatory cytokines, interleukin 6 (IL-6), monocyte chemoattractant protein-1 (MCP-1), tumor necrosis factor (TNF) and zinc-α2-glycoprotein (AZGP1) and pancreatic enzymes have been shown to be related to CAC [14, 20–23]. Clinical evidence had shown that IL-6 in tumor biopsy tissue is an inflammatory marker in the diagnosis of CAC, and high levels of IL-6 in tumor tissue and serum correlate with weight loss and muscle atrophy [24, 25]. IL-6 is a pleiotropic proinflammatory cytokine secreted by normal monocytes, fibroblasts, endothelial cells, Th2 cells and vascular endothelial cells. A variety of tumor cells also secrete IL-6 [14, 20, 26], which can target macrophages to regulate the tumor microenvironment [14, 27]. CAC is a classic metabolic syndrome of type 2 diabetes with insulin resistance, and muscular dystrophy is a hallmark of CAC [28]. In addition, over-expression of IL-6 aggravates weight loss of cancer cachexia in Apc^Min/+^ mice, whereas increased knockout of IL-6 reverses that [29]. Multiple studies have suggested that IL-6 leads to insulin resistance and promotes muscle atrophy, as skeletal muscle is the main tissue in which insulin stimulates glucose uptake [30–32]. In short, various studies have proven that IL-6 can induce insulin resistance, and as a consequence indirectly exacerbates muscle loss in CAC.

In our study, the data shows that the crosstalk between cancer cells and macrophages in muscle tissues is that tumor cells secrete IL-6 inducing macrophages to up-regulate MMP12. It may provide a new point view of MMP12 function in weight loss induced by CAC.

## Methods

### Mice

B6.129X-MMP12tm1Sds/J macrophage metalloelastase-deficient (MMP12^−/−^) mice, with a C57BL/6 background were purchased from The Jackson Laboratory, USA (No.T001457, https://www.jax.org/strain/004855). All Apc^Min/+^ mice (No. T001457) were obtained from Gem Pharmatech, China (No. 004855, http://www.gempharmatech.com/cn/index.php/searchinfo/59/17.html). Wild-type (WT/C57BL/6 J) mice were purchased from Guangdong Medical Laboratory Animal Center, China, the production license number is SCXK (Guangdong) 2017-0125. Apc^Min/+^; MMP12^−/−^ hybrid mice were obtained by hybridizing Apc^Min/+^ mice with MMP12^−/−^ mice (Fig. S1A). All mice were housed under specific-pathogen-free conditions. All animal studies were complied with Guangdong Pharmaceutical University, and all protocols were approved by the Animal Experimental Ethics Committee of Guangdong Pharmaceutical University.

### Genotype identification

Male Apc^Min/+^ mice were hybridized with female C57 mice and bred to create Apc^Min/+^; MMP12^−/−^ hybrid mice. Genotype identification were performed on the 3-week-old mice The PCR products were subjected to gel electrophoresis (1.2%), and a gel imaging system (GboxGyngene system, UK) was used to obtain electrophoresis images (Fig. S1B). Details of genotype identification can be found on the website of The Jackson Laboratory or Gem Pharmatech, China (850 point mutation).

### Mouse experiments and clinical human tissue collection

Bone marrow, blood, inguinal white adipose tissue (iWAT), interscapular brown adipose tissue (BAT), gastrocnemius and soleus muscles were collected after euthanizing mice with carbon dioxide.

For immunohistochemistry staining analysis, muscle tissues were obtained from the residual tissue of patient who underwent clinical trauma surgery. All clinical fresh blood samples were collected from the Cancer Center of Sun Yat-sen University in Guangzhou, China, including healthy individuals who served as controls and patients with CRC (30-60 years old, excluding patients with diabetes and hyperthyroidism) in the experimental group.

### Antibodies

Anti-F4/80 (Cat.:14-4801-81) and anti-MMP12 (Cat.: MA5-24851) were purchased from eBioscience and Thermo Fisher, respectively. Anti-GAPDH (Cat.: 5174P) and anti-β-actin (Cat.: 4970S) were purchased from Cell Signaling Technology Inc. (CST). Recombinant mouse MMP-12 protein (Cat.: 3467-MPB-020) was purchased from R&D Systems, Inc. Alexa Fluor-488 donkey antibody (Cat.: P/N SA11055S) was purchased from Thermo Fisher Scientific, Cambridge, Massachusetts, USA.

### Cell culture

RAW264.7, MC38 and CT26 cell lines were purchased from American Typical Culture Collection (ATCC) and cultured according to international standard protocols. All cell lines were maintained in Dulbecco’s Modified Eagle’s Medium (DMEM, Thermo Scientific HyClone, Beijing, China) + 10% fetal bovine serum (HyClone) + 1% penicillin/streptomycin (HyClone) and cultured in DMEM. All cell lines in the experiments were incubated at 37 °C, 5% CO_2_.

### Total RNA extraction and real-time PCR

All tissues from mice were stored at − 80 °C until dissolved in Trizol. RNA extraction was performed according to the manufacturer’s instructions, and the total extracted RNA was reverse-transcribed into cDNA for PCR amplification using the real-time polymerase chain reaction SYBR Green kit (TaKaRa, China). Steps of PCR were as follows: denaturation at 94 °C for 5 min; 40 cycles of denaturation at 94 °C for 30 s, annealing at 60 °C for 30 s, and extension at 72 °C for 30 s; extension at 72 °C for 5 min. The mRNA samples were quantified in triplicate. The housekeeping gene GAPDH was used as an internal control to normalize the real-time PCR data for each mRNA sample. All real-time PCR primers were synthesized by Shanghai Sangon Biotechnology Inc., China, and the primer sequences are listed in S Table 1.

### Histological analysis and Hematoxylin and eosin staining

All tissues were fixed with 10% neutral buffer formalin and embedding in paraffin after dehydration. The 3-μm tissue sections were treated with hematoxylin eosin, immunohistochemical staining and immunofluorescence staining, respectively. In this study, muscle fiber area was assessed using imageJ software after hematoxylin and eosin staining. ImageJ software was used to analyze color statistics of the cross-sectional area. Muscle area was selected after setting the threshold.

### Immunohistochemistry

Tissue sections were dewaxed and incubated with 3% hydrogen peroxide in methanol and blocked with 10% bovine serum albumin diluted with phosphate buffered saline (PBS). The sections were then incubated with primary antibodies at 4 °C overnight. After treated with horseradish-peroxidase-conjugated secondary antibody (1:100), the sections were color development with DAB and then stained with hematoxylin for microscopic observation. PBS as the negative control.

### Dual immunofluorescence staining

Tissue sections were dewaxed and blocked with 10% bovine serum albumin in PBS solution. The sections were incubated with a mixture of primary antibodies (anti-MMP12 antibody, 1:100, and anti-F4/80 antibody, 1:100) overnight at 4 °C. The primary antibody-treated sections were then incubated with a mixture of secondary antibodies (conjugated Alexa Fluor 488, 1:100, and Alexa Fluor 555, 1:100) for 1 h at room temperature. Immunostaining signals and DAPI-stained nuclei were visualized under a confocal microscope.

### ELISA assay

ELISA was performed to test serum samples from patients and mice following the manufacturer’s protocol. The human-IL-6 kit (Cat.EHC007), mouse JE/MCP1/CCL2 kit (Cat.EMC113), mouse IL-6 ELISA kit (Cat.EMC004), and mouse KC/IL-8/CXCL1 ELISA kit (Cat.EMC104) were purchased from NeoBioscience Technology Company (ShenZhen, China). The rat/mouse insulin kit (Cat.EZRMI-13 K) was purchased from EMD Millipore Corporation. Mouse MMP12 ELISA kit (Cat.ARG81803, Arigo Biolaboratories) and human CXCL1/KC kit (Cat. EK-196, Multi Science Company). ELISA data were analyzed by Curve Expert 1.4 software.

### Western blot

Tissue samples (50-80 μg) and cells were homogenized and lysed with radioimmunoprecipitation assay buffer (Thermo Scientific, Cat.: 89900) containing protease and phosphatase inhibitors, and then centrifuged to collect supernatants. Quantitative analysis based on the bicinchoninic acid (BCA) protein assay was used to determine protein concentration. Denatured proteins were separated by sodium dodecyl sulfate–polyacrylamide gel electrophoresis (10% SDS-PAGE) and transferred to polyvinylidene difluoride (Millipore Corporation, Billerica, MA, USA) membranes, blocked with 5% nonfat milk, and then incubated with primary antibodies overnight at 4 °C. Next day, the protein strips were further incubated with horseradish-conjugated secondary antibodies (1:5000) and the bands were developed with enhanced chemiluminescence detection solutions. ImageJ software was used to analyze optical density of the bands. All experiments were repeated three times.

### Oral glucose tolerance test (OGTT), insulin tolerance test (ITT) and blood glucose level measurement

OGTT: After fasting for 8 h, the mice were given 2 g of glucose per kilogram of body weight orally. Fasting blood and then blood samples were collected at 30, 60, 90, and 120 min, respectively. ITT: After fasting for 8 h, the mice were intraperitoneally injected with 0.75 IU of insulin per kilogram of body weight. Fasting blood and then blood samples were collected at 30, 60, 90, and 120 min, respectively. The whole blood glucose level is measured at the tail vein using a blood glucose meter.

### Serum lipid assay

Levels of total cholesterol (TC), total triglycerides (TG), high density lipoprotein cholesterol (HDL-C) and low density lipoprotein cholesterol (LDL-C) were determined according to the manufacturer’s protocols. All assay kits were purchased from Jiancheng Biotech (Nanjing, China).

### Cytokine Array

The culture media with MC38 or non-MC38 cells was detected with Ray Bio Mouse Cytokine Antibody Array 5 (Cat.: AAM-INF-1-2, 38 cytokines, Ray Biotech) according to manufacturer’s protocol. In brief, the membranes were blocked by incubated with the blocking buffer. Diluted biotin-conjugated anti-cytokine antibodies and HRP-conjugated streptavidin were used to develop positive signal. The visualized X-ray film was exposed to enhanced chemiluminescence (ECL) for quantification. Data analysis was performed using ImageJ to determine signal intensity, and positive controls were used to normalize the data. Each ratio of cytokine density to positive control density represents the relative content of every cytokine. The cytokines and their abbreviations are shown in Fig. S7.

### Co-culture experiment

All cells were grown in a mixture of DMEM + 10% FBS + 10% penicillin-streptomycin. The co-culture of RAW264.7 and MC38/CT26 cells were seeded in 6-well dishes (Corning, NY, USA) using a chamber with filter inserts (pore size: 0.4 μm). None of the cell lines could pass through the filter because the pore size of the filter was smaller than the diameter of the cell lines. RAW264.7 cells without co-cultured MC38/CT26 cell lines (−MC38/CT26) were used as negative controls. MC38/CT26 cell lines (positive controls, 1 × 10^4^, 3 × 10^4^, 5 × 10^4^) were seeded in the upper chamber, while RAW264.7 cells (negative controls, 1-2 × 10^5^) were seeded in the lower chamber. We then physically separated RAW264.7 cells from MC38/CT26 cell lines to obtain RAW264.7 cells in the lower chamber. The RAW264.7 cells were homogenized and lysed with immunoprecipitation buffer for quantitative analysis and then subjected to western blot.

### Macrophages treated with IL-6

IL-6 (Cat. No.: 216-16, PeproTech) was dissolved in aqueous solutions of trehalose plus bovine serum albumin (BSA). RAW264.7 cells (1-2 × 10^5^) were seeded in 6-well plates and treated with increased doses of IL-6 (0, 2, 5 10, 30 ng/ml) for 72 h. Cells incubated with fresh media without IL-6 were used as the negative controls (−IL-6). Finally, western blot was used to quantify MMP12 in RAW264.7 cells under different conditions.

### Isolation of primary peritoneal macrophages

24-week-old WT and Apc^Min/+^ mice were sterilized with 75% ethanol after cervical dislocation. The mouse abdomen was opened from the peritoneum, and 5 mL fetal bovine serum was injected using a syringe. After 5 min, the peritoneal fluid was collected and transferred to a 15 mL tube to obtain peritoneal macrophages. After centrifuging (1000 rpm) for 10 min and removing the supernatant, the pelleted cells were resuspended in DMEM and then cultured at 37 °C for 2 h.

### MMP12 and peptide interaction experiments

Recombinant mouse MMP-12 protein (Cat. No.:3467-MPB-020) was purchased from R&D Systems, Inc. Following the manufacturer’s instructions, MMP12 was dissolve at a concentration of 250 μg/ml in a pH 7.5 buffer containing 50 mM Tris, 10 mM CaCl_2_, 150 mM NaCl, 0.05% (w/v) Brij-35, and 5 μM ZnCl_2_. Insulin polypeptide and insulin-like factor polypeptide were synthesized by ChinaPeptides (Shanghaih, China).

The sequence of insulin and IGF-1 are 5-FAM-NQHLCGSHLVEALYLVCGERGFFYTPK (Dabcyl) and 5-FAM-GPETLCGAELVDALQFVCGDRGFYFNK (Dabcyl), respectively. The peptide freeze-dried powder was dissolved in 25% ACN and 75% ddH_2_O solvent at a concentration of 1 mg/ml. For fluorescence intensity test, after mixed incubation of MMP12 and peptide (37 °C, 2 h), the fluorescence intensity was measured using a fluorescence microplate reader. For IMS Analysis, after MMP12 and peptide were mixed and incubated, the lower liquid filtered by a 34KD filter was subjected to Electrospray Ionization Mass Spectrometry (ESI-MS) to detect its characteristic peaks, and the experimental conditions were: Ion Source: ESI, Capillary (KV): ± 2500 ~ 3000, Desolvation (L/hr): 800, Desolvation Temp: 450 °C, Cone(V): 30 ~ 50, Run Time: 1 min.

### Mice administration

MMP408 (MMP12 inhibitor, Cat.: 444291, Merck), CAS 1258003-93-8, could control the biological activity of MMP-12 [33, 34]. All 17-week-old Apc^Min/+^ mice were randomly divided into three groups (*n* = 5 per group). One group of Apc^Min/+^ mice were intragastrically administered with MMP408 at a dose of 5 mg/kg, and the other group was intraperitoneally administered with 5-FU (30 mg/kg) combined with a dose of MMP408 by intragastric administration. Meanwhile, Apc^Min/+^ mice injected with normal saline as the control. Body weight was measured after the administration every 2 days, which continued for 10 days. Weight changes were recorded.

### Data processing

All mouse organ ratios were presented by the percentage of the organs/tissue weight to the body weight. The ratio of the skeletal muscle weight to the body weight was equal to (Gastrocnemius +Soleus muscle)/body (%). All data were processed by using GraphPad Prism 8.0 software and were presented as the means ± standard deviations (SD). A two-tailed test or a one-way ANOVA test was used to analyze the data. Statistically significant differences were considered at *P < 0.05, **P < 0.01, ***P < 0.001, ****P < 0.0001. The schematic images were created on BioRender.com.

## Results

### MMP12 knockout leads to weight gain in Apc^Min/+^ mice

**Fig. 1:**
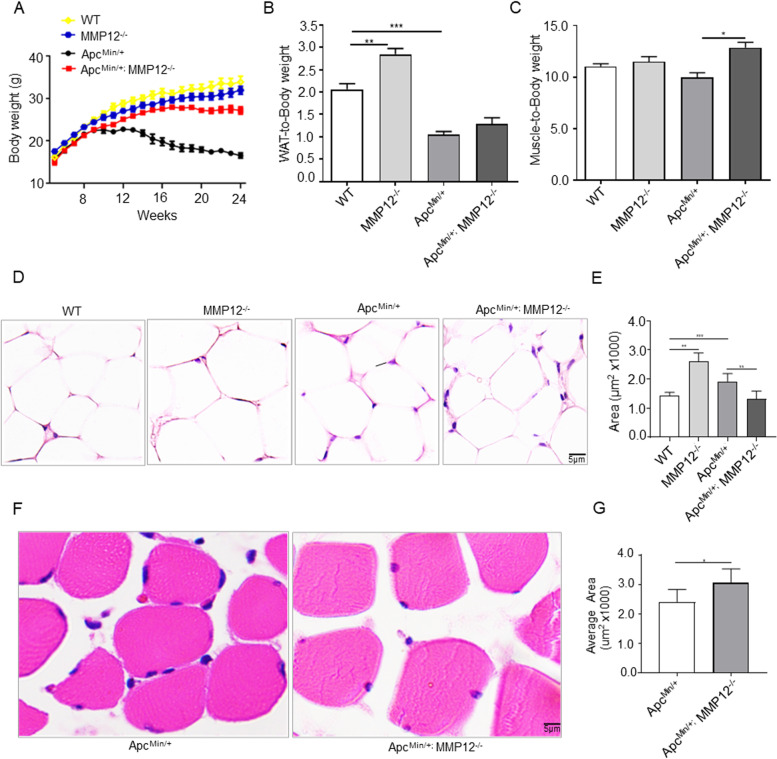
Knockout of MMP12 in Apc^Min/+^ Mice Prevents Weight and Muscle Loss. **A** Plots of the body weight of wild-type (WT), Apc^Min/+^, Apc^Min/+^; MMP12^−/−^ and MMP12^−/−^ mice from 5 to 24 weeks (*n* = 6 per group). **B** The ratio of inguinal white adipose tissue to body weight (***P < 0.001; **P < 0.01, *n* = 5). **C** The ratio of skeletal muscle to body weight (*P < 0.05, *n* = 5). **D** Hematoxylin and eosin staining of inguinal white adipose tissue in WT, Apc^Min/+^, Apc^Min/+^; MMP12^−/−^ and MMP12^−/−^ mice at 24 weeks old. Scale bars: 5 μm. **E** Evaluation of the inguinal white adipose tissue across 4 groups by ImageJ software (40X) (***P < 0.001; **P < 0.01, Each cell area are shown as means ± SD; *n* = 5 mice per group). **F** Hematoxylin and eosin staining of muscle in Apc^Min/+^ and Apc^Min/+^; MMP12^−/−^ at 24 weeks old. Scale bars: 5 μm. **G** Evaluation of the cross-sectional area of the gastrocnemius from Apc^Min/+^ mice and Apc^Min/+^; MMP12^−/−^ mice by ImageJ software (40X) (*P < 0.05; data are shown as means ± SD; *n* = 6 mice per group)

To investigate body weight change in wild type mice (WT mice) or Apc^Min/+^ mice after MMP12 knockout, we determined their body weight from 5 to 24 weeks old. The weight curves showed that compared with weight gain in the WT mice 5 to 24 weeks, the body weight of Apc^Min/+^ mice reached its maximum peak at 15 weeks old and then declined until the mice were sacrificed at approximately 24 weeks old (*P* < 0.5). Surprisingly, in comparison with the Apc^Min/+^ mice group, the body weight of Apc^Min/+^; MMP12^−/−^ hybrid mice was increased by approximately 70% at the same age (*P* < 0.01). Meanwhile, there were no significant difference in the body weight between WT mice and MMP12^−/−^ mice (*P* > 0.5, Fig. 1A). Apc^Min/+^ mouse model present cancer cachexia (CAC) like human being with tumor, with intestinal tumor burden and weight decrease dramatically. Since weight loss induced by CAC may be related to the wasting of skeletal muscle weight and fat weight [30], we hereby investigated whether weight gain in Apc^Min/+^mice caused by MMP12 knockout was due to a reduction in fat and skeletal muscle loss at 24 weeks old. The histological WAT of Apc^Min/+^mice and compared with that of WT mice. The WAT-to-body weight ratio of Apc^Min/+^ mice was decreased; the ratio of Apc^Min/+^; MMP12^−/−^ mice tended to increase compared with that of Apc^Min/+^ mice, but the increase was not statistically significant (Fig. 1B). However, a significant increase of approximately 4.5% in the muscle-to-body weight ratio was observed in Apc^Min/+^; MMP12^−/−^ mice compared with Apc^Min/+^ mice (Fig. 1C). To further confirm the histological changes in WAT (Fig. 1D) and muscle area (Fig. 1F) of the four mice group at 24 weeks old, the hematoxylin and eosin staining (H&E staining) was used to assess the histological area by ImageJ software. We observed that the fat area was larger in MMP12^−/−^ mice compared with that in WT mice, but it the fat area was reduced between Apc^Min/+^ mice and Apc^Min/+^; MMP12^−/−^ mice (Fig. 1E). While H&E staining of muscle suggested that the area in Apc^Min/+^; MMP12^−/−^ mice was estimated to be approximately 1.2-fold larger than that in Apc^Min/+^ mice (Fig. 1G). Meanwhile, no difference in food intake was observed between Apc^Min/+^and Apc^Min/+^; MMP12^−/−^ mice (Fig. S3A). Taken together, we think that knocking out MMP12 leads to weight gain and prevents muscle wasting in Apc^Min/+^ mice.

### MMP12 is up-regulated in muscle tissue and peritoneal macrophages of Apc^Min/+^ mice

**Fig. 2:**
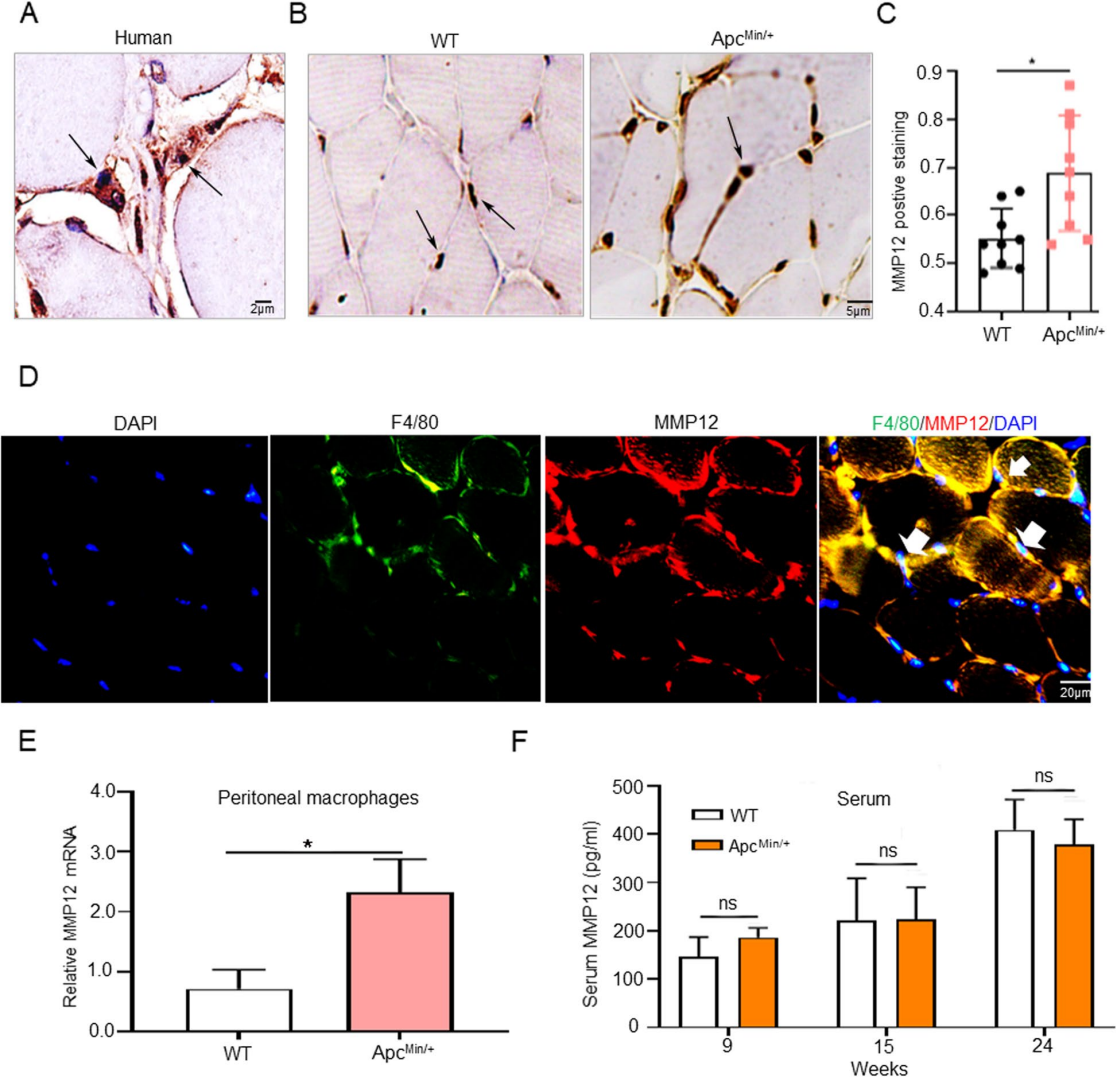
MMP12 Is Up-regulated in Muscle Tissues and Macrophages of Apc^Min/+^ Mice. **A** MMP12 antibody immunostaining in the muscle of healthy individuals. Scale bar: 2 μm. **B** Immunostaining of MMP12 in muscle tissue of WT mice and Apc^Min/+^ mice. Scale bar: 5 μm. **C** Quantification of MM12 expression in gastrocnemius tissue was performed by ImageJ software (40X) (*P < 0.05, data are shown as means ± SD; *n* = 9 per group). **D** Representative images of dual immnofluorescent staining of macrophages (F4/80 in green) and MMP12 (in red) in WT mice are shown. The yellow areas in the merged images indicate overlapping localization of the red and green signals, indicated by the white arrows. Scale bars: 20 μm. **E** Quantification of MMP12 mRNA level in peritoneal macrophages isolated from WT mice and Apc^Min/+^ mice by qPCR (*P < 0.05; data are shown as the means ± SD; *n* = 6 per group). **F** The serum MMP12 levels detected in WT and Apc^Min/+^ mice at 9, 15, and 24 weeks old by ELISA (*P* > 0.05; data are shown as means ± SD; *n* = 6 per group)

Based on the results above, we used immunohistochemical staining, immunofluorescence staining and western immunoblotting to detect and confirm whether MMP12 was expressed in muscle tissue. The immunohistochemistry results showed that MMP12 positive staining was expressed not only in skeletal muscles in human being (Fig. 2A and Fig. S3B), but also in mice (Fig. 2B). To further explore why muscle loss caused in Apc^Min/+^ mice but not in WT mice by knocking out MMP12, we used immunohistochemistry to detect MMP12 in the muscle of 24-week-old WT mice and Apc^Min/+^mice, respectively (Fig. 2B). Immunohistochemical staining results showed that MMP12-positive staining was increased in the muscle tissue of Apc^Min/+^ mice compared with that of WT mice at 24 weeks old (Fig. 2C). MMP12 is mainly secreted by macrophages [15], we next performed dual immunofluorescence (IF) to determine whether MMP12 and macrophages were co-expressed in muscle tissues, and results indicated that F4/80 (macrophage cell marker) and MMP12 protein co-localized in muscle tissues (Fig. 2D). Next, Quantitative PCR (qPCR) revealed that a tendency towards higher MMP12 mRNA levels in peritoneal macrophages (as described in the Materials) was observed in the Apc^Min/+^ mice (Fig. 2E), which was consistent with the immunohistochemistry results. Then MMP12 levels in serum were detected with ELISA kit, the data showed that they were not significantly different between the 9-, 15-, and 24-week-old WT mice and the Apc^Min/+^mice (Fig. 2F). In summary, MMP12 was co-expressed with macrophage in muscle tissue. MMP12 was increased in skeletal muscle tissue and peritoneal macrophages of Apc^Min/+^ mice but in serum. In addition we have also evaluated the mRNA expression of MMP12 in other tissues (Fig. S3C, obtained from The Cancer Genome Atlas).

### IL-6 are increased in tumor cells, serum and Colon Cancer tissue

**Fig. 3:**
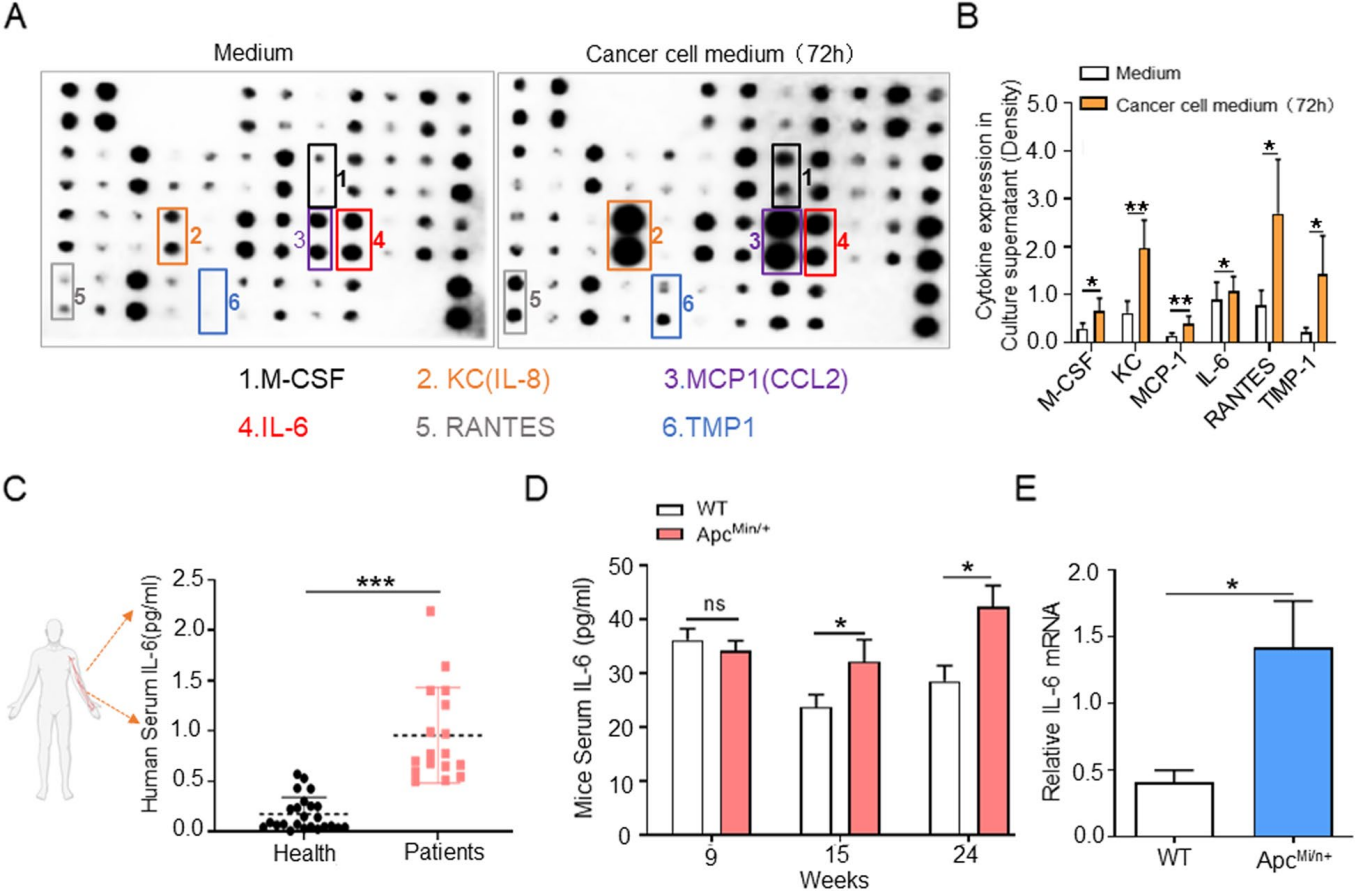
Tumor Cells Are An Source of IL-6. **A** Cytokine array was used to detect untreated medium and MC-38 cells medium after 72 h; arrows indicate the significantly increased cytokines. **B** The relative quantification of the significantly up-regulated cytokine to positive quality control density ratio by ImageJ software. The positive quality control density was determined for normalization (**P < 0.01, *P < 0.05; data are shown as means ± SD). **C** Serum interleukin 6 (IL-6) levels in normal individuals and patients with colorectal cancer aged 30-50 years detected by ELISA (***P < 0.001; data are shown as means ± SD; Health group: *n* = 26; Patients group: *n* = 18). **D** The IL-6 levels in serum in WT mice and Apc^Min/+^mice were detected at 9, 15, and 24 weeks old by ELISA (*P < 0.05; data are shown as means ± SD; *n* = 5 per group). **E** IL-6 mRNA expression was validated in normal intestinal epithelium isolated from Apc^Min/+^ mice versus that in intestinal tumors of WT mice by qPCR (*P < 0.05; data are shown as means ± SD; *n* = 4 per group)

Previous studies have shown that IL-6 is one of the predictors of CAC-induced muscle atrophy [75-78], which may aggravate the disease development. IL-6 in tumor biopsy tissue is a biomarker for the diagnosis of cancer cachexia, and tumor cells are an important source of IL-6 [20, 26, 35]. Clinical literature also suggested that IL-6 is almost the only cytokine that increases among various factors in many patients with CAC muscle atrophy who lose weight. Therefore, we focused on investigating whether IL-6, which is associated with muscle atrophy and cancer cells. Cytokine microarray was used to determine which cytokines were up-regulated in the supernatant of MC38 mouse colon cancer cell line. Previous studies have reported that both MC38 and CT26 mouse colon cancer cells can up-regulate IL-6 expression [36]. In vitro protein microarray experiments showed that IL-6 expression was higher in the supernatant after cultured with MC-38 cells (Fig. 3A, B). Hence, we focus on studying IL-6. Clinical data also showed that serum IL-6 in colorectal cancer patients were significantly higher than the healthy group (Fig. 3C). A similar trend was also found in Apc^Min/+^ mice serum that IL-6 levels in Apc^Min/+^ mice were significantly increased compared with that in WT mice at 15 to 24 weeks old (Fig. 3D). We also detected by qPCR that the IL-6 mRNA levels was higher in the stripped intestinal tumor tissues of Apc^Min/+^mice than in the normal intestinal epithelium of WT mice (Fig. 3E). Taken together, serum IL-6 was up-regulated in colorectal cancer patients and in Apc^Min/+^ mice, and IL-6 was increased in both colorectal cancer tissues and cells.

### IL-6 up-regulate MMP12 of macrophages

**Fig. 4:**
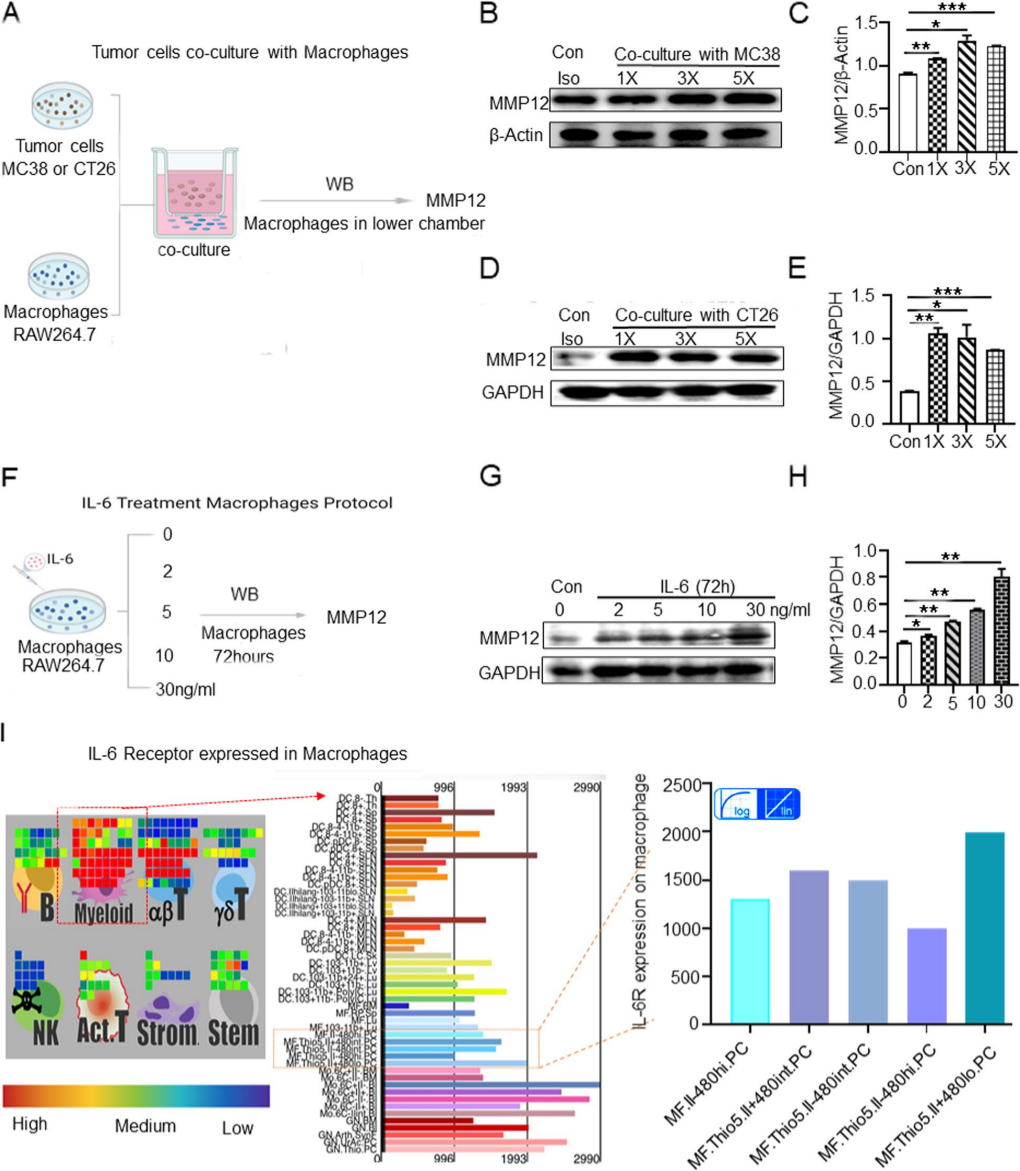
IL-6 Up-regulates MMP12 in Macrophages. **A** Schematic diagram of tumor cells (MC38/CT26 cell lines) co-cultured with macrophage cells (RAW264.7 cell lines). All quantifications were performed by image J software for gray scale statistics. **B**, **C** Representative western blots showing the secreted MMP12 protein levels from RAW264.7 cell lines (1-2 × 10^5^) cultured alone or co-cultured with MC38 cell lines (control, 1 × 10^4^, 3 × 10^4^, 5 × 10^4^). β-Actin as the internal control. **D**, **E** Representative western blots showing the secreted MMP12 protein levels from RAW264.7 cell lines (1-2 × 10^5^) cultured alone or co-cultured with CT26 cell lines (control, 1 × 10^4^, 3 × 10^4^, 5 × 10^4^). GAPDH as the internal control. **F** Schematic diagram of IL-6 treated macrophages. RAW264.7 cells incubated with fresh media were served as untreated negative controls. Western blot was used to detect MMP12 in RAW 264.7 cells and GAPDH as the internal control. **G**, **H** RAW264.7 cells (1-2 × 10^5^) were seeded in 6-well plates and treated with increasing doses of IL-6 (0, 2, 5, 10, 30 ng/mL) for 72 h. **I** Immune Gene data (https://www.immgen.org/ImmGenApps.html) suggest that IL-6 receptor expresses on F480+ macrophages. The colored bars refer to the expression level of IL-6 receptors on macrophages. Red represents high expression of IL-6 receptors, and green represents low expression of IL-6 receptors

Previous studies reported that IL-6 might not directly lead to muscle loss in CAC [26, 37]. IL-6 in the tumor microenvironment are an important determinant of alternative macrophage activation and could induce macrophage M2 polarization, and M2 macrophages can produce MMP12 [38, 39]. Taken together, we speculated that tumor cells release IL-6 to stimulate macrophages to up-regulate MMP12. In order to reveal the potential association between tumor-derived IL-6 and macrophages, we performed that mouse macrophage RAW264.7 cells were co-cultured with mouse colorectal cancer MC38 cells / CT26 cells, after 72 h to detect MMP12 in macrophages by western blot (Fig. 4A). Results showed that RAW264.7 cells exhibited increased MMP12 expression as the number of MC38 cells increased, compared with RAW264.7 cells cultured alone as the negative control group (Fig. 4B, C). Similar trends were observed in CT26 cells (Fig. 4D, E). We further treated RAW264.7 cells with IL-6 using different concentrations. RAW264.7 cells were seeded in 6-well plates and treated with increasing doses of IL-6 (0, 2, 5, 10, 30 ng/ mL) for 72 h. Cells incubated with fresh media were used as the untreated negative controls (Fig. 4F). For RAW264.7 cells treated with IL-6, we assessed MMP12 levels by western blot. We found that within a certain concentration range (< 30 ng/ml), as the IL-6 dose was increased, MMP12 expression in RAW264.7 cells was also elevated when treated with IL-6 (Fig. 4G, H). Meanwhile, immune gene data suggested that IL-6 receptor (IL-6R) was highly expressed on myeloid cells including F480^+^ macrophages (Fig. 4I). Altogether, these findings indicate that IL-6 can stimulate macrophages and up-regulate MMP12 in macrophages.

### MMP12 degrade insulin and insulin-like growth Factor-1

Above results suggest that IL-6 can up-regulate MMP12 in macrophages. MMP12 knockout can reduce muscle loss in Apc^Min/+^ mice. It is important that insulin and insulin-like growth factor 1 (IGF-1) have complex anabolic effects and are important regulators of muscle remodeling that can mediate muscle atrophy [40–44]. Moreover, Jung-Ting Lee proposed that MMP12 expression significantly promotes insulin resistance and that insulin were regulated by resident macrophages [15]. Hereby we hypothesized that MMP12 may degrade insulin or IGF-1 which could impact muscle loss as previously reported [17].

**Fig. 5:**
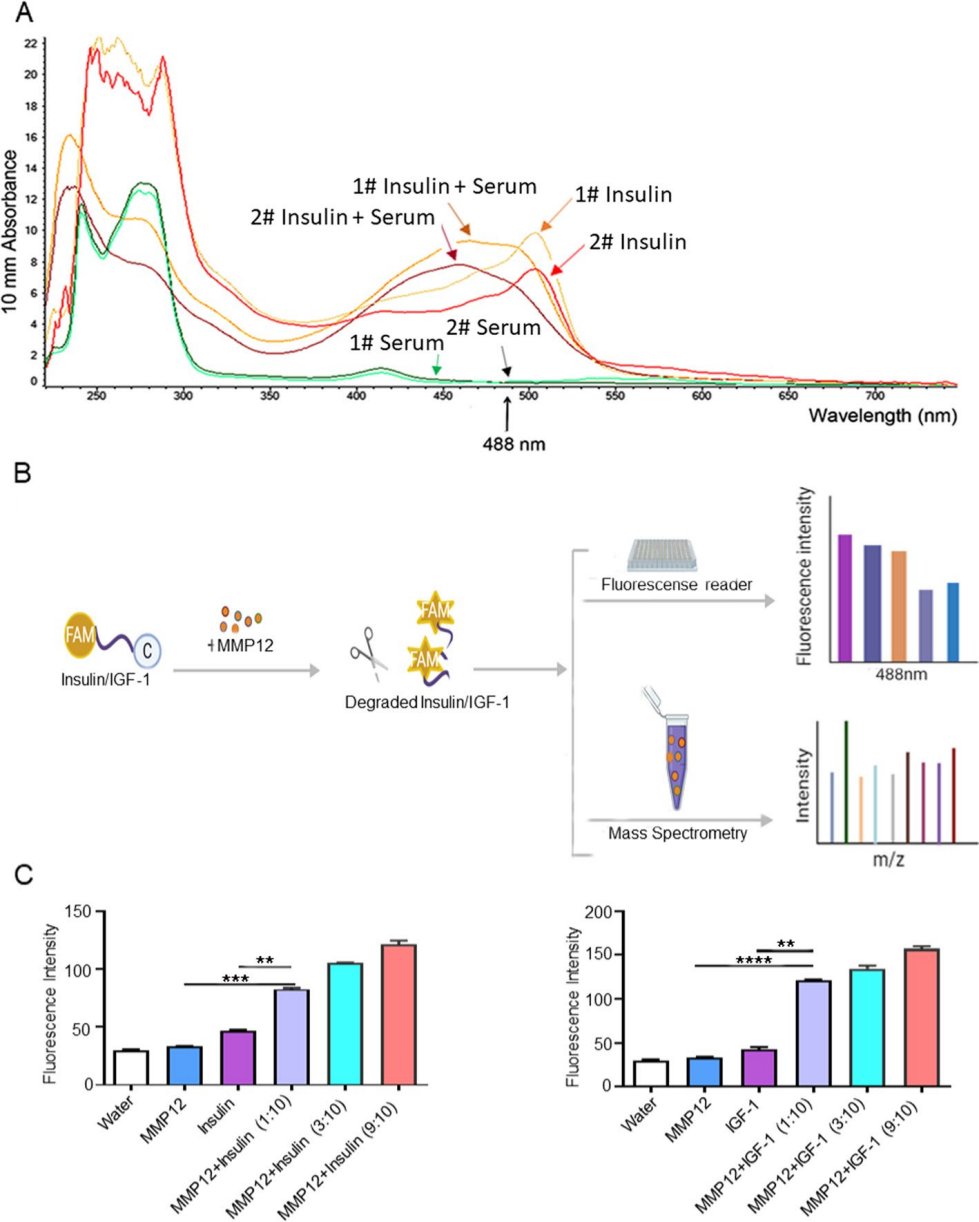
MMP12 Degrades Insulin and Insulin-like Growth Factor-1. **A** Representative images of the peak were detected at 488 nm after the insulin peptide was degraded by normal mice serum. The insulin peptide was labeled with FAM (488 nm) and DABCLY (quenching fluorescence). When the insulin peptide was broken, FAM was then observed at 488 nm (resonance energy transfer, FRET). **B** The synthetic insulin (or IGF-1) peptide was labeled with FAM and DABCLY as shown. The insulin peptide was labeled with FAM (488 nm) and DABCLY (quenching fluorescence). When the insulin (IGF-1) peptide was broken, FAM was then observed at 488 nm. Fluorescence intensity of the peak were detected at 488 nm after the insulin (IGF-1) peptide was degraded by MMP12 using a fluorescence microplate reader. Electrospray Ionization Mass Spectrometry (IMS) was used to detect its characteristic peaks. **C** The MMP12 and insulin (or IGF-1) peptide interaction led to appearance of a fluorescence signal on dose-dependent (***P < 0.001, **P < 0.01; data are shown as means ± SD)

Firstly, we labeled insulin polypeptide with FAM and DABCLY on the N side and the C side. The insulin peptide was labeled with FAM (488 nm) and DABCLY (quenching fluorescence). When the insulin peptide was broken, FAM was then observed at 488 nm (resonance energy transfer, FRET). Then we found that  the insulin peptide incubated it with seum, with the absorbance peak appearing at λ = 488 nm (Fig. 5A). Because IGF-1 is similar to insulin in structure, we further evaluated the relationship between IGF-1and MMP12, and measured its fluorescence intensity and characteristic peak at 488 nm (Fig. 5 A,B). The dose of insulin and IGF-1 fluorescent peptide was constant with MMP12, the more MMP12 protein was present, the stronger the fluorescence intensity was, as shown in Fig. 5C. Results of electrospray ionization mass spectrometry (IMS) showed that after incubated with MMP12 protein, insulin was decomposed into different fragments (Fig. S2A), and its characteristic peak changed from (m/z = 436.99) to various m/z characteristic peaks. Similarly, IGF-1 was cleaved into fragments with different m/z after incubated with MMP12, and its characteristic peak (m/z = 436.98) was also altered (Fig. S2B). We hypothesized that insulin and IGF-1 might be broken down into different amino acid groups by MMP12. Totally, MMP12 may degrade insulin and IGF-1, and it seems that the degradation is stronger on IGF-1 than on insulin.

In addition, our other data supports that knocking out MMP12 in Apc^Min/+^ mice may reduce insulin levels or increase insulin sensitivity, which could contribute to reversing insulin resistance (Fig. S6A-H), while it was not related to the basic function of islets based on results of H&E staining and IHC staining (Fig. S4E-H).

### MMP12 inhibitor reverses weight loss in Apc^Min/+^ mice

**Fig. 6:**
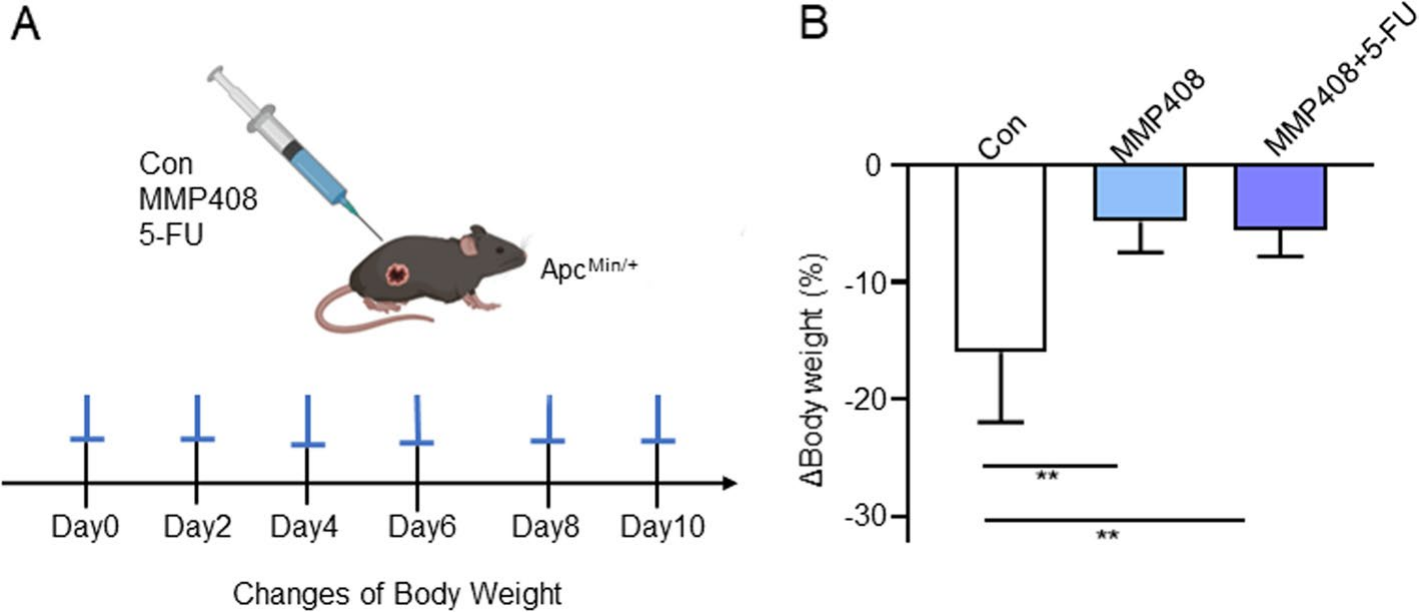
Inhibiting MMP12 in Apc^Min/+^ Mice Slows Down Weight Loss. **A** Schematic diagram of the administration process of 17-week-old Apc^Min/+^ mice. The drugs were given every 2 days (MMP408: 5 mg/kg, 5-FU: 30 mg/kg). The saline group was used as a control. **B** Percentage of weight gain compared to the basal weight after administration of drugs in Apc^Min/+^ mice (**P < 0.01, data are shown as means ± SD; *n* = 5 per group)

The insulin and IGF-1 can impact muscle loss caused by CAC and exacerbate weight loss [40, 43–45]. Therefore, we wonder whether MMP12 inhibitor would influence weight changes in Apc^Min/+^ mice. To investigate the effect of inhibiting MMP12 on colorectal cancer or body weight, we administered MMP12 inhibitor (MMP408) and its combination with a classic clinical anti-colon cancer drug (5-FU) in Apc^Min/+^ mice (Fig. 6A). After 2 weeks of administration, results showed that in 17-week-old Apc^Min/+^ mice, weight loss in the MMP12 inhibitor group (MMP408 group) accounted for 5% of the basal body weight, which was only one third of that of the control group (administered with normal saline), indicating a significant difference between the two groups. In the experiment group of combined MMP408 and anticancer drug (+MMP408/+ 5-FU), weight change was decreased by approximately 8% of the basal body weight, which was half that of the control group (+Control). Unfortunately, there were no changes in body weight in the anticancer drug combination group (+MMP408/+ 5-FU), when compared with those in the MMP12 inhibitor group alone (+MMP408) (Fig. 6B). In summary, the experiments above suggested that specifically inhibiting MMP12 in Apc^Min/+^ mice can reduce weight loss.

**Fig. 7:**
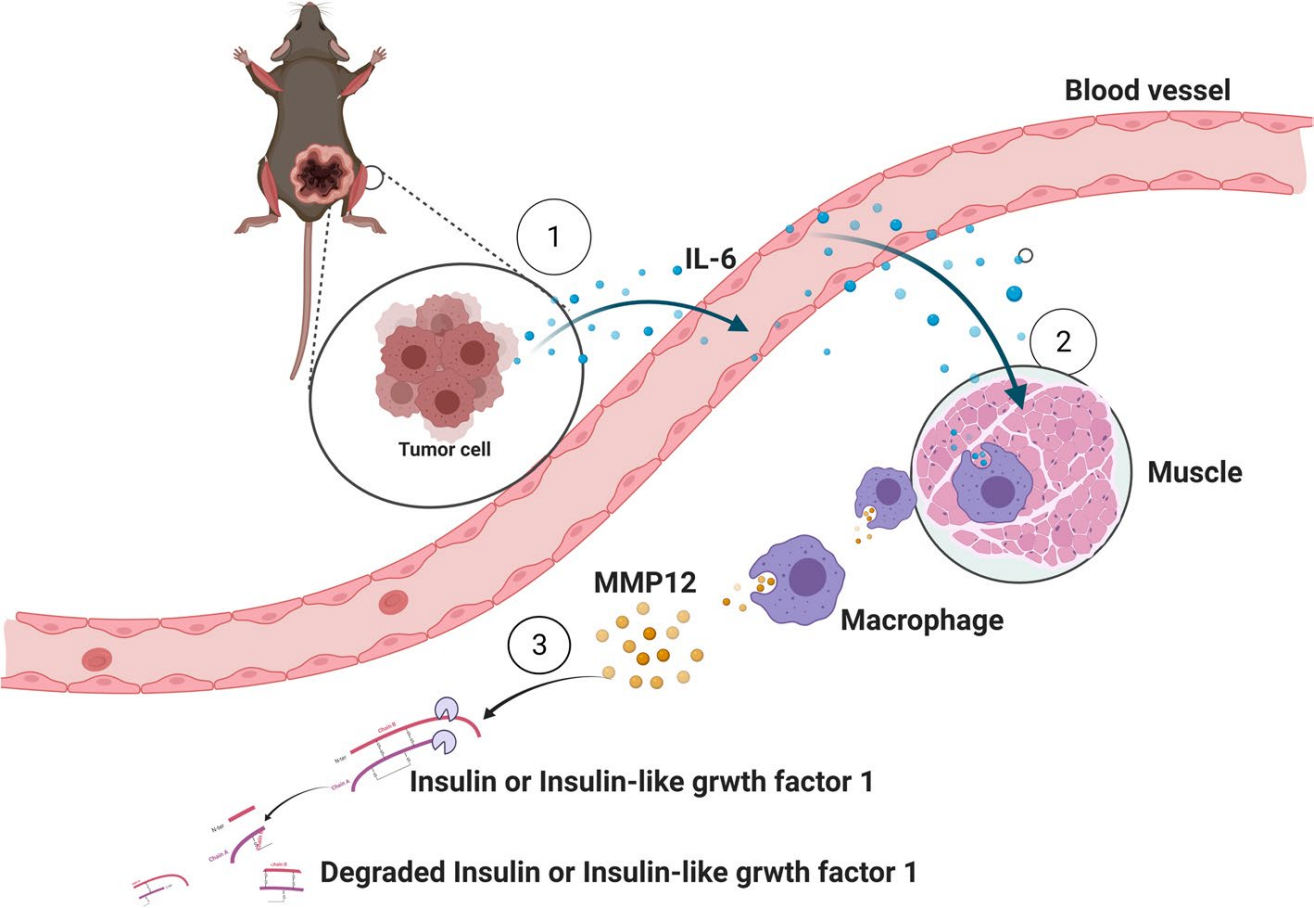
The Schematic of the Crosstalk between Tumor Cells And Muscular Macrophages in Apc^Min/+^ Mice via IL-6 And MMP12

Lastly, we think that it exist the crosstalk between cancer cells and macrophages in muscle tissues, it is that tumor cells secrete IL-6 inducing macrophages to up-regulate MMP12 which degrade insulin and IGF-1 in muscle tissue (Fig. 7).

## Discussion

Weight loss has received more and more attention in various diseases, such as diabetes, abnormal thyroid metabolism, weight control, cancer, etc. More than four-fifths of patients with CAC died from extreme loss of body weight and skeletal muscle. Our study suggests that MMP12 plays a new role in controlling weight and muscle loss in Apc^Min/+^ mice, and inhibiting MMP12 can reverse the body weight reduction associated with CAC in mice.

The clinical definition of CAC by Fearon criteria includes the following characteristics: weight loss > 5% or weight loss > 2% and a BMI < 20 kg/m^2^ or sarcopenia [46]. In this study, 15- to 24-week-old Apc^Min/+^ mice with weight loss > 15%, as well as skeletal muscle (gastrocnemius and soleus) loss, and certain other symptoms such as anemia, were considered as the characteristics of CAC. The data of this study demonstrated that muscle weight and muscle cross-sectional area were increased in Apc^Min/+^ mice. The data of Apc^Min/+^; MMP12^−/−^ mice, compared with those of Apc^Min/+^ mice, indicated that knocking out MMP12 might suppressed body weight and skeletal muscle loss. Our previous study suggested that MMP12 knockout would increase tumor growth by impacting macrophage development, while there was no difference in the survival rate [22, 47], which can be explained by the protective effect of MMP12 knockout on the weight loss of tumor-bearing mice.

In our study, we weighed these mice tissue at 24 weeks old, and performed histological evaluation using H&E staining. We found that knocking out MMP12 had some additional effect on weight of liver, BAT, and pancreas in Apc^Min/+^ mice. Similarly, knocking out MMP12 in Apc^Min/+^ mice did not cause changes in WAT, even though MMP12 knockout in WT mice resulted in increased and expanded WAT, which is in agreement on Lee Jung-Ting’s findings [15].

IL-6 is mainly secreted by a variety of immune cells and is also highly expressed in a variety of cancer cells [48]. Our study suggested that MC38 cell lines can secrete IL-6. In vivo animal experiments showed that IL-6 in the serum of Apc^Min/+^ mice was higher than that in the serum of WT mice at 15 weeks and 24 weeks old, respectively, which is consistent with the findings of a previous study [11]. IL-6 mRNA levels in intestinal tumors were increased compared with those in normal intestinal epithelial tissue, which echoes the data of increased serum IL-6 in clinical cancer patients [49]. The cytokines secreted by MC38 cells certainly also include MCP1 and KC, which can recruit macrophages [50–52]. However, our experiments have shown that increased MCP1 mRNA levels was only found in intestinal tumors (Fig. S5A) but not in the serum of Apc^Min/+^ mice at the CAC stage (15 - 24 weeks) (Fig. S5B). There was no difference in serum KC ((Fig. S5E) (Fig. S5D) and KC mRNA levels(Fig. S5C) in late-stage tumors in mice. Similarly, in clinical studies, serum KC did not differ between normal healthy individuals and patients with colorectal cancer (Fig. S5E). So MCP1 and KC were not being explored more in our study.

As known that knocking out IL-6 could reduce muscle consumption in Apc^Min/+^ mice [11]. The crosstalk between tumors and inflammatory cytokines is also well known [22, 29]. Here the results show that IL-6 would induce macrophage secreting MMP12. However, the limitation of our preliminary work is that the relationship between tumor-derived IL-6 and macrophage MMP12 has not been well explored. It is very important to specifically inhibit MMP12 in Apc^Min/+^ mice to reduce weight loss, Another, we did not explore if other cytokines were secreted by tumors or macrophages, which would have effect on MMP12 secretion by. This should be done more in that point.

We used fluorescence electrospray ionization mass spectrometry methods to confirm that insulin and IGF-1 were degraded by MMP12, but the specific sites of amino acids in insulin and IGF-1 have not been explored. MMP12 degrades insulin in our study, which is supported by the findings of a previous study [17]. Our data and other study indicated that MMP12 was closely related to glucose and lipid metabolism, resulting in loss of skeletal muscle and adipose tissue [40–44]. Taken together, results suggest that MMP12 is closely related to glucose metabolism in Apc^Min/+^ mice. We think that knocking out MMP12 in Apc^Min/+^ mice may lead to partly insulin degradation, and affect insulin sensitivity and the balance of glucose utilization induced by tumor growth. Four kinds of mice blood lipid levels also showed that when had MMP12 knock out in Apc^Min/+^ mice, total triglycerides were decreased in the early and middle stages, and high density lipoprotein cholesterol was increased in all age groups, while total cholesterol and low density lipoprotein cholesterol did not change among the 4 groups shown in Fig. S6I-L.

One question is that if knockout MMP12 would reverse muscle loss under condition of cachexia. Previous Study showed that long-term treatments with high-dose IL-6 may cause additional side effects, such as exacerbating CAC which will result in more muscle loss [53]. Here we find that MMP12, a downstream factor of IL-6, MMP12 inhibitor could significantly suppress weight loss in mice, although such effect was not enhanced after combined treatment with 5-FU (chemotherapy drug). Our study provides a new insight for the clinical treatment of cancer cachexia.

## Conclusions

MMP12 may play a positive role in the process of glucose metabolism, lipid metabolism and cancer-induced cachectic muscle loss. To our knowledge, this is the first study to investigate the effect of glucose and lipid metabolism on body weight in Apc^Min/+^; MMP12 knockout mice, establishing a relationship between tumor cell-derived IL-6 and MMP12 of macrophages. To sum up, our study demonstrate that knocking out MMP12 in Apc^Min/+^ mice significantly reduced muscle loss. There is a correlation between tumor-derived IL-6 and macrophage MMP12 in colorectal cancer. MMP12 can degrade insulin and IGF-1, MMP12 knockout has a great impact on glucose metabolism and lipid metabolism. Therefore, inhibiting MMP12 may represent a new potential target for the clinical treatment of cancer patients with weight loss.

## Supplementary Information

**Additional file 1.**

**Additional file 2.**

## Data Availability

The related data and material in the section of my manuscript is available. When reasonably requested, the data set used and/or analyzed in the current study can be obtained from the author. The data sets supporting the results of this article are included within the article and its additional files.

## Abbreviations

MMP12: Matrix Metalloproteinases 12
CAC: Cancer Cachexia
Apc^Min/+^; MMP12^-/-^: Apc^Min/+^; MMP12 knockout mice
iWAT: Inguinal White Adipose Tissue
BAT: Brown Adipose Tissue (interscapular)
HRP: Horseradish Peroxides
H&E: Hematoxylin & Eosin
qPCR: Quantitative Polymerase Chain Reaction
IHC: Immunohistochemistry
IF: Immunofluorescence
PBS: Phosphate Buffered Saline
MCP1(CCL2): Monocyte Chemoattractant Protein 1
KC(CXCL1): Keratinocyte-derived Chemokine
RANTES: Regulated upon Activation, Normal T Cell Expressed and Presumably Secreted
IL-6: Interleukin 6
IGF-1: Insulin-like Growth Factor 1
