# Supplementary material for: MMP12 knockout prevents weight and muscle loss in tumor-bearing mice
Source: BMC Cancer. 2021 Dec 4;21:1297. doi: 10.1186/s12885-021-09004-y (PMC8642861; doi:10.1186/s12885-021-09004-y)
Supplement: Supplementary file 1 — Additional file 1. [file 12885_2021_9004_MOESM1_ESM.docx]

**Supplementary data**

**
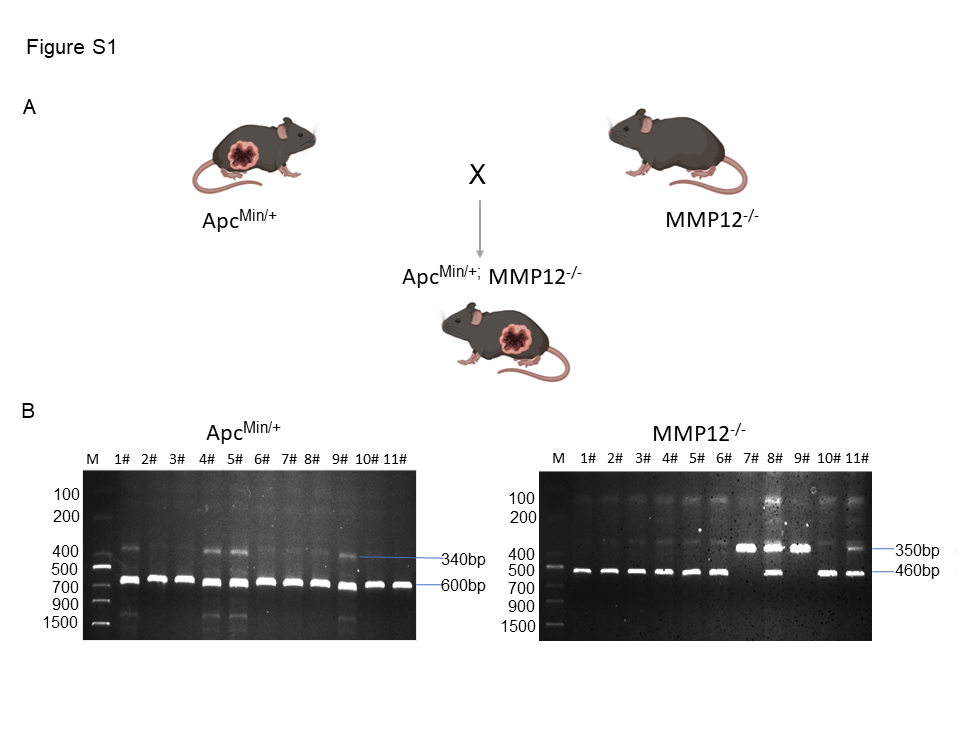
**

**[Figure S1](#s) Mouse crossbreeding and genotype identification**

(A) The schematic of crossbreeding Apc^Min/+^ mice with MMP12^-/-^ mice to obtain Apc^Min/+^; MMP12^-/-^ mice. (B) The APC mutant gene PCR product size was 340 bp, while the PCR product size in wild-type (WT) mice was 600 bp. The MMP12 knockout (mutation) PCR product size was 460 bp, while the PCR product size in WT mice was 350 bp. Specifically, Apc^Min/+^: 1#, 4#, 5#, 9#; MMP12^-/-^: 1#, 2#, 3#, 4#, 5#, 6#, 10#; Apc^Min/+^; MMP12^-/-^: 1#, 4#, 5#.

**
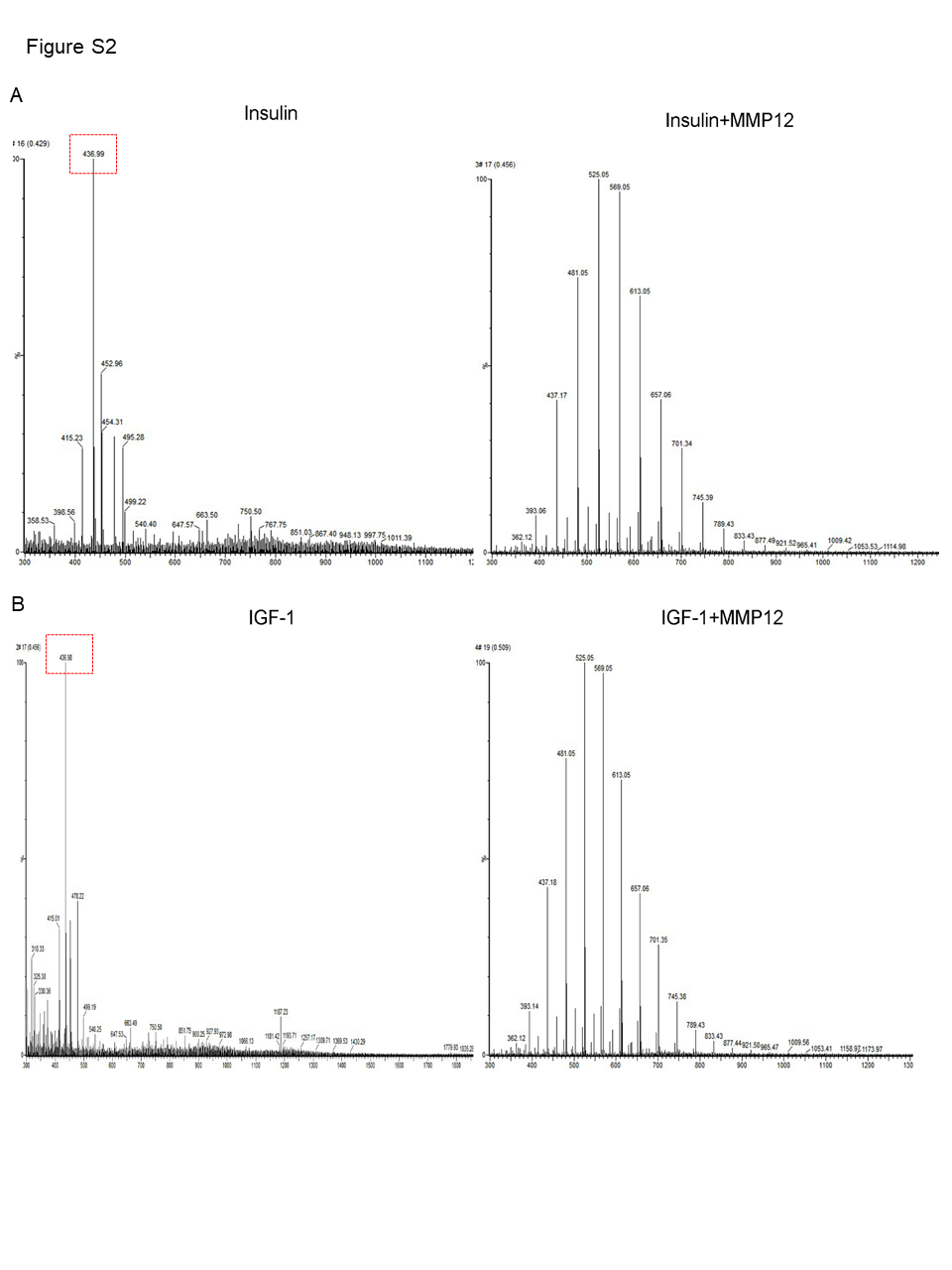
**

**[Figure S2](#ss) Electrospray ionization mass spectrometry (ESI-MS) analysis report: The characteristic ion peaks of insulin precursor and insulin-like growth factor 1 (IGF-1) precursor were fragmented into fragment ion peaks of different m/z after incubated with MMP12 protein, respectively.**(A) The results of ESI-MS showed that after incubated with MMP12 protein, the characteristic ion peak of insulin precursor (m/z = 436.99) was fragmented into fragment ion peaks of different m/z. (B) The characteristic ion peak of IGF-1 precursor (m/z = 436.98) was fragmented into fragment ion peaks of different m/z after incubated with MMP12.

**
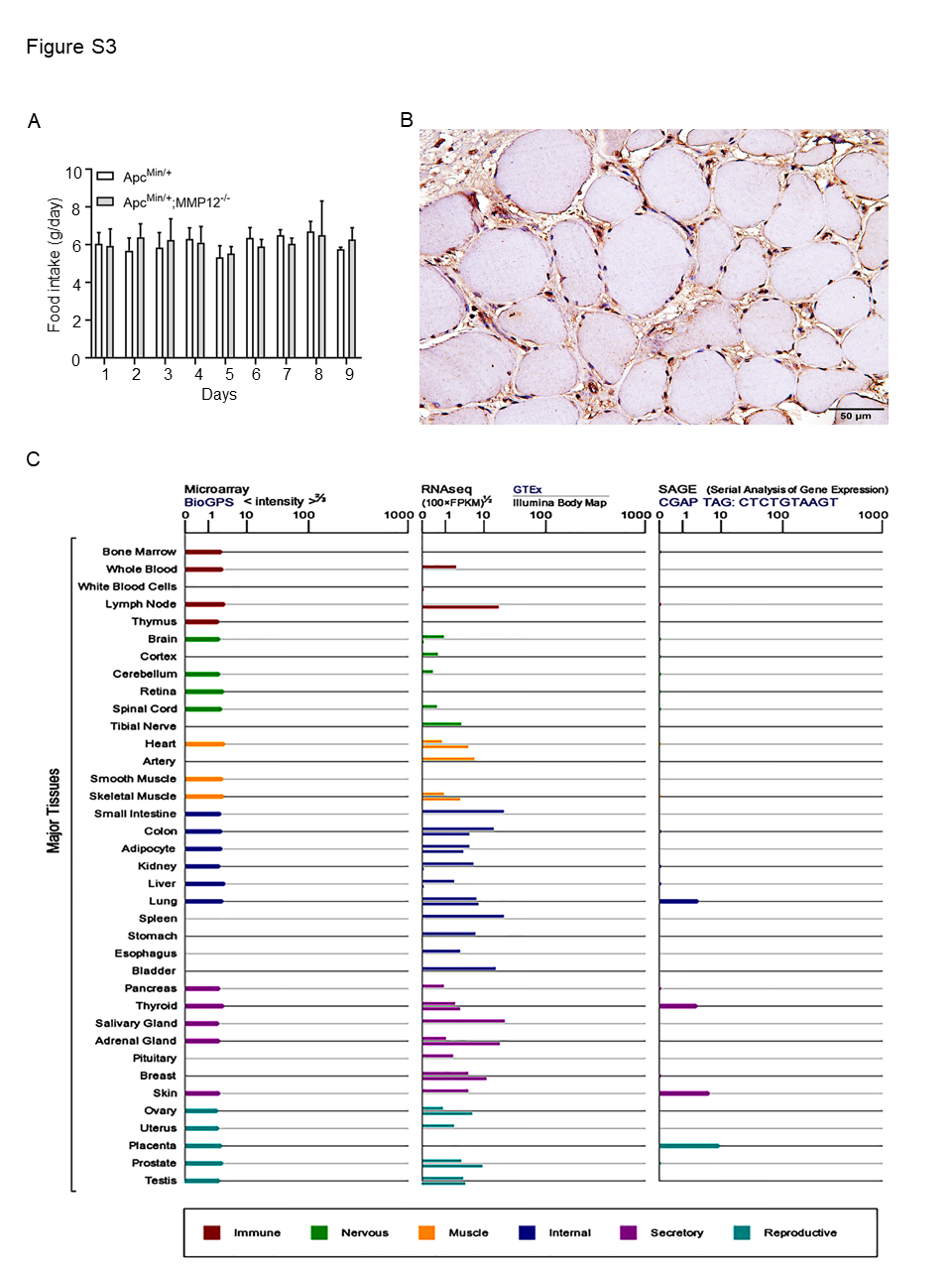
**

[**Figure S3**](#sss) **MMP12 knockout did not significantly affect food intake in Apc^Min/+^ mice, and MMP12 was expressed in bone marrow, muscle, liver and fat tissues, etc.**

1. There was no statistical difference in food intake between Apc^Min/+^ mice and Apc^Min/+^; MMP12^-/-^ mice. The weight of food consumed after fasting for 8 h was measured every day starting from the 17^th^ week. Each group of mice were fed in three cages, and each cage held 5 mice. (B) Immunostaining of MMP12-positive in gastrocnemius muscle from the clinical individual. Scale bar: 50μm. (C) Analysis of MMP12 mRNA data obtained from The Cancer Genome Atlas (TCGA), for GTEx, Illumina, BioGPS and SAGE of MMP12 gene in normal human tissues.


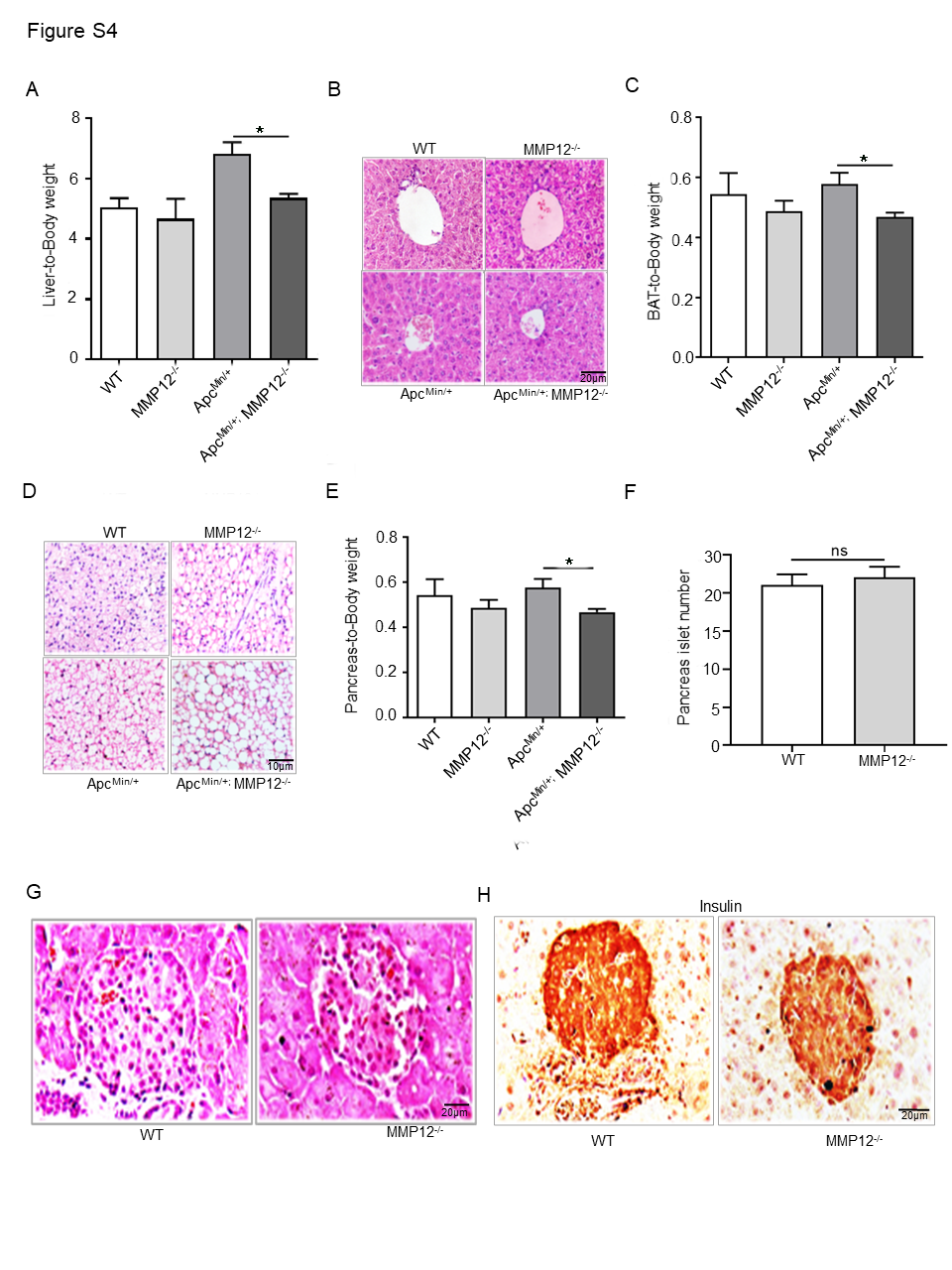


**[Figure S4](#ssss) The effect of knocking out MMP12 on liver, brown adipose tissues and pancreatic islets in Apc^Min/+^ mice**

(A) The liver-to-body weight ratio (data are shown as means ± SD; n = 6 per group; **P*< 0.05) (B) H＆E staining of the liver at 24 weeks. Scale bar: 20μm (data are shown as means ± SD; n = 6 per group; **P*< 0.05). (C) The brown adipose tissue (BAT)-to-body weight ratio was higher in Apc^Min/+^; MMP12^-/-^ mice than that in Apc^Min/+^ mice (data are shown as means ± SD; n = 6 per group; **P*< 0.05). (D) Results of H＆E showed that white fat increased in brown adipose tissue in MMP12 knockout mice. Scale bar: 10μm. (E) The pancreas-to-body weight ratio (data are shown as means ± SD; n = 6 per group; **P*< 0.05). (F) The number of islets in WT and MMP12^-/-^ mice (data are shown as the means ± SD; n = 6 per group; *P*> 0.05). (G) H＆E staining of the pancreas in WT and MMP12^-/-^ mice at 24 weeks. Scale bar: 20μm. (H) Immunostaining of insulin in the pancreas of WT and MMP12^-/-^ mice at 24 weeks. Scale bar: 20μm.


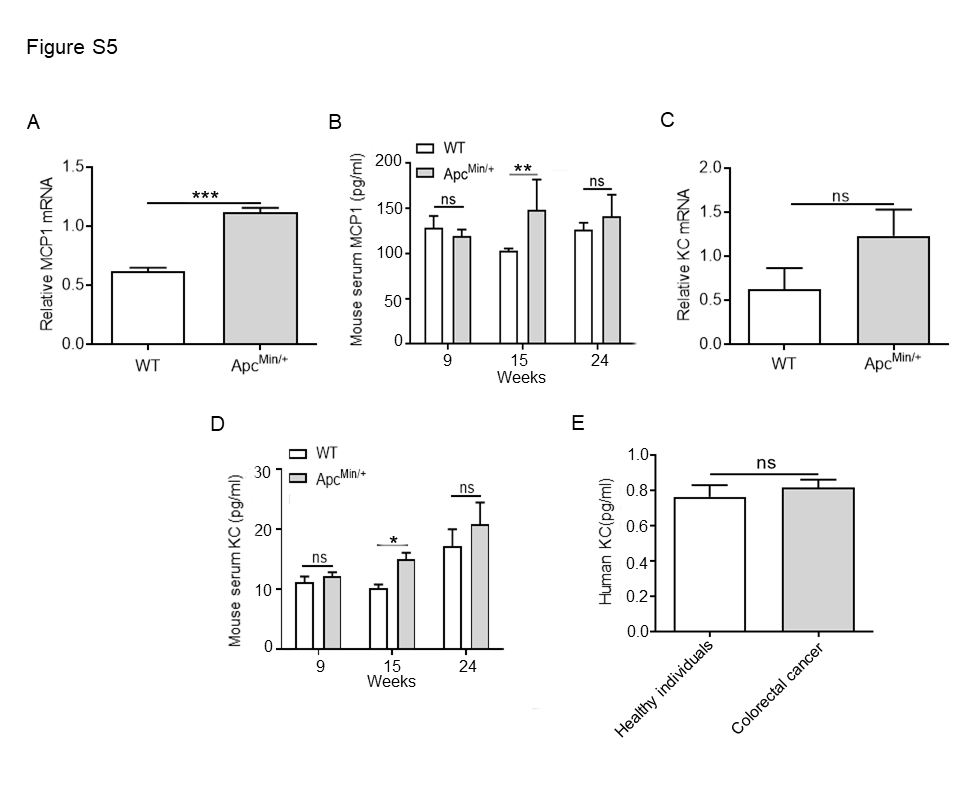


[**Figure S5**](#sssss) **Serum monocyte chemoattractant protein 1（MCP1）and keratinocyte-derived chemokine (KC) showed no statistical difference in Apc^Min/+^ mice at 24 weeks.**

**(**A, C) The mRNA expression of MCP1 and KC was validated by qPCR in normal intestinal epithelium isolated from WT mice compared with that in intestinal tumors isolated from Apc^Min/+^ mice (data are shown as means ± SD; n = 4 per group; ****P<*0.001). (B, D) Serum MCP1 and KC in Apc^Min/+^ mice versus in WT mice at 9, 15 and 24 weeks (data are shown as means ± SD; n = 6 per group; ***P<* 0.01, **P*< 0.05, respectively). (E) Serum KC in normal healthy individuals and colorectal cancer patients detected by ELISA (data are shown as the means ± SD; n = 6 per group; *P*> 0.05).


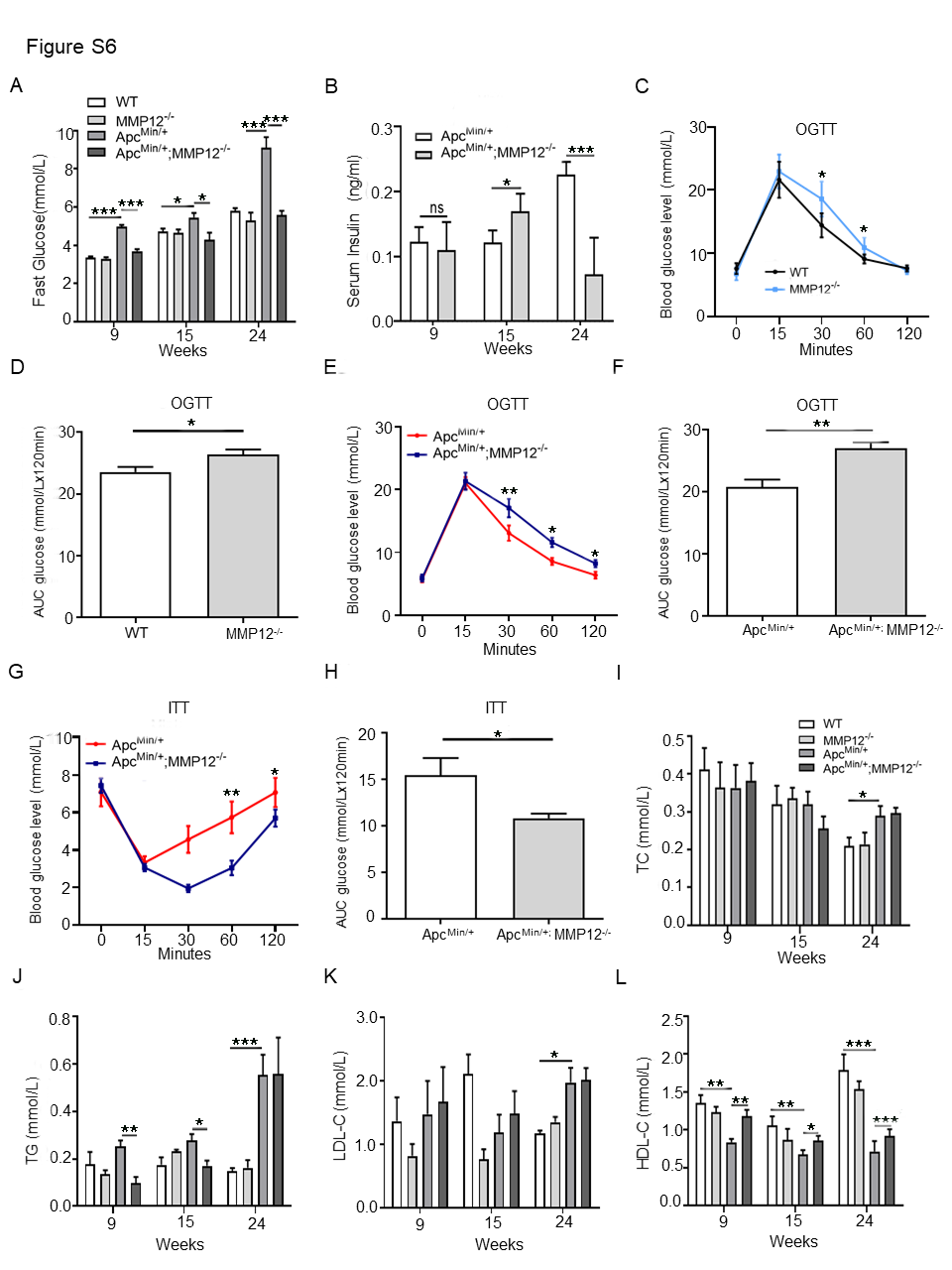


[**Figure S6**](#ssssss) **MMP12 Knockout present glucose and lipid metabolism changes in Apc^Min/+^ mice.**

1. Fasting plasma glucose levels at 9, 15 and 24 weeks in WT mice, MMP12^-/-^mice, Apc^Min/+^ mice, and Apc^Min/+^; MMP12^-/-^ mice (data are shown as the means ± SD; n = 4 per group; ****P<*0.001, **P*< 0.05, respectively). (B) Fasting serum insulin levels were detected by ELISA in Apc^Min/+^ and Apc^Min/+^; MMP12^-/-^ mice at approximately 9, 15 and 24 weeks (data are shown as the means ± SD; n = 4 per group; ****P<*0.001; **P<* 0.05, respectively). (C) Oral Glucose Tolerance Test (OGTT): WT and MMP12^-/-^ mice were fasted for 4 h and then administered with glucose (75 IU/kg). Fasting blood then blood samples were collected at 30, 60, 90, and 120 minutes, respectively (data are shown as the means ± SD; n = 6 per group; **P<* 0.05). (D) Area under the curve (AUC) analysis for OGTT revealed greater AUC in MMP12^-/-^ mice compared with that in WT mice (data are shown as means ± SD; n = 6 per group; **P*< 0.05). (E) OGTT: Apc^Min/+^ and Apc^Min/+^; MMP12^-/-^mice were fasted for 4 h and then administered with glucose (75 IU/kg). Fasting blood then blood samples were collected at 30, 60, 90, and 120 minutes, respectively (data are shown as means ± SD; n = 6 per group; ***P<* 0.01, **P*< 0.05, respectively). (F) AUC analysis of OGTT revealed a significant increase in Apc^Min/+^; MMP12^-/-^ mice compared with that in Apc^Min/+^mice (data are shown as means ± SD; n = 6 per group; ***P<* 0.01). (G) Insulin tolerance test (ITT): Apc^Min/+^ and Apc^Min/+^; MMP12^-/-^mice received an ip injection of insulin. Fasting blood then blood samples were collected at 30, 60, 90, and 120 minutes, respectively (data are shown as means ± SD; n = 6 per group; ***P<* 0.01). (H) AUC analysis of OGTT, which was significantly decreased in Apc^Min/+^; MMP12^-/-^ mice compared with that in Apc^Min/+^ mice (data are shown as means ± SD; n = 6 per group; **P*< 0.05). (I-L) Quantitative determination of serum total cholesterol (TC), total triglyceride (TG), low density lipoprotein-cholesterol (LDL-C), high density lipoprotein-cholesterol (HDL-C) by kits in WT mice, MMP12^-/-^ mice, Apc^Min/+^ mice and Apc^Min/+^; MMP12^-/-^ mice at 9, 15 and 24 weeks (data are shown as means ± SD; n = 7 per group; ****P<*0.001, ***P<* 0.01, **P*< 0.05, respectively). Details of OGTT and ITT are as described in the Materials.

**
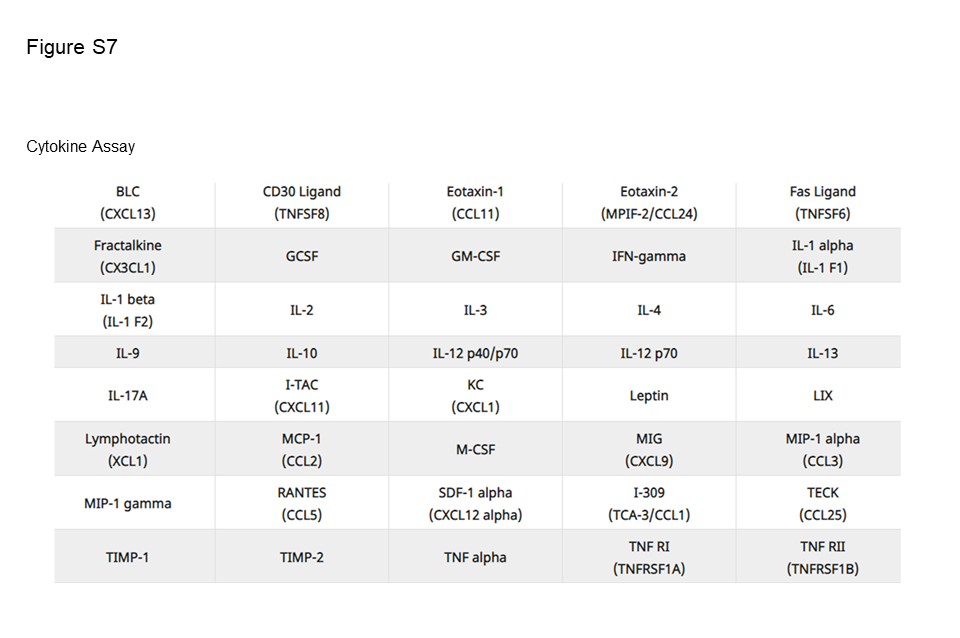
**[**Figure S7**](#sssssss) **Cytokine array kit contains 40 kinds of cytokines.**


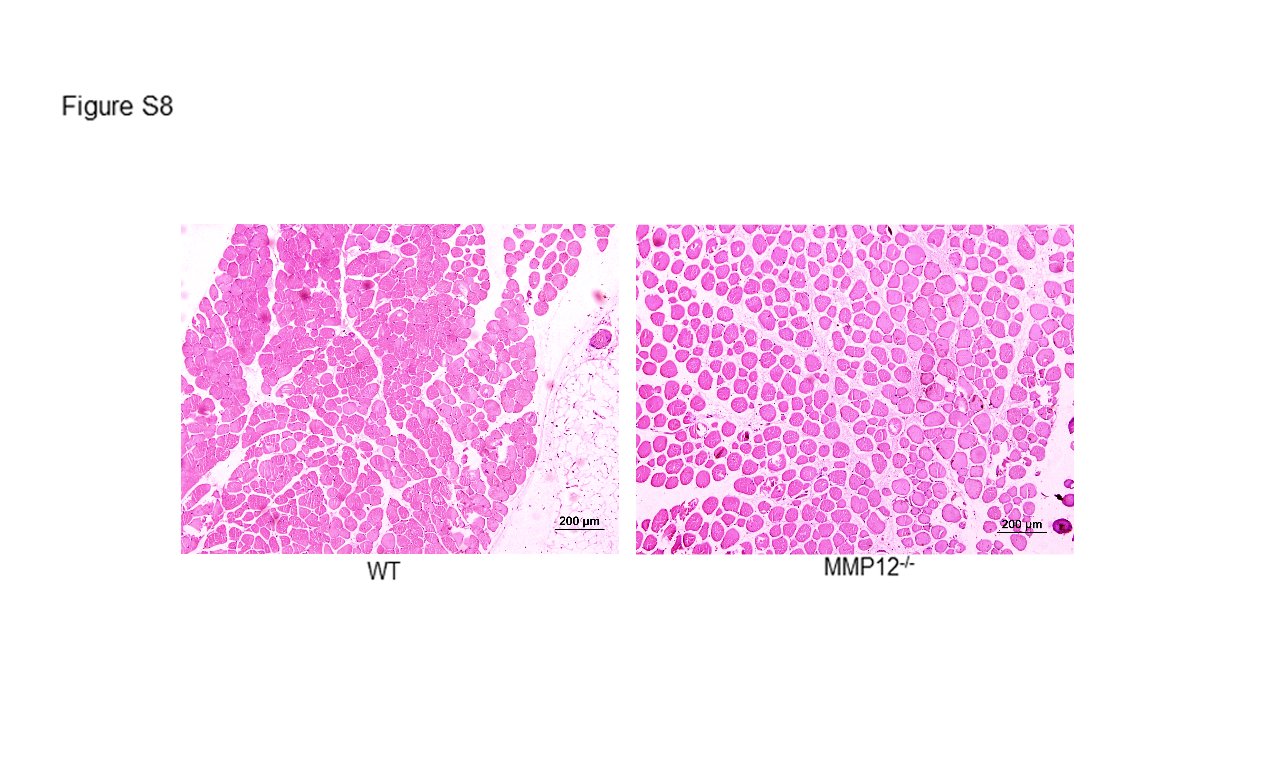


[**Figure S8**](#sssssss) **H&E staining of muscle in WT and MMP12^-/-^ mice at 24 weeks. Scale bar: 200μm.**

**[Table1](#T): List of Quantitative PCR primers.**

| S [Table1](#T) List of quantitative PCR primers | | | |
| --- | --- | --- | --- |
| Gene Name | Accession Number | Forward (5'‑3') | Reverse (5'‑3') |
| m-MMP12 | nm_001320076.1 | GAGTCCAGCCACCAACATTAC | GCGAAGTGGGTCAAAGAC |
| m-GAPDH | nm.001289726.1 | CGTCCCGTAGACAAAATGGT | TCAATGAAGGGGTCGTTGAT |
| m-IL6 | nm.31168 | TAGTCCTTCCTACCCCAATTTCC | TTGGTCCTTAGCCACTCCTTC |
| m-MCP1(CCL2) | nm.11333 | TTAAAAACCTGGATCGGAACCAA | GCATTAGCTTCAGATTTACGGGT |
| m-KC(CXCL1/IL8) | nm.008176 | CTGGGATTCACCTCAAGAACATC | CAGGGTCAAGGCAAGCCTC |
| Note: m, mouse. | | | |

[**Table 2**](#TT) **The relationship between IL-6 and body weight were reported in the following references**

| S [Table 2](#TT) | | |
| --- | --- | --- |
| Various studies on IL-6 | Highlights of Impact on Body Weight (Fat or Muscle) | PMID |
| Overexpression of IL-6 | Circulating IL-6 levels were increased and the animals lost both weight and body fat rapidly (often when mice bore tumors); Chronically elevated IL-6 levels led to hyperinsulinemia, reduced body weight, impaired insulin-stimulated glucose uptake by the skeletal muscles. Systemic IL-6 overexpression in tumor-bearing Apc^Min/+^ mice accelerated cachexia development, which coincided with suppressed basal and eccentric contraction-induced muscle protein synthesis. | [18437347](https://pubmed.ncbi.nlm.nih.gov/18437347/);  [29641213](https://pubmed.ncbi.nlm.nih.gov/29641213/) |
| IL-6 Inhibited (Inhibit the STAT3/JAK/IL-6R) | Mice body weight were increased to resist cancer cachexia. Therapeutic effects of IL-6R blockade on promoting muscle regeneration. IL-6R blockade has therapeutic effects on the dystrophic skeletal muscle. | [31002945](https://pubmed.ncbi.nlm.nih.gov/31002945/);  [11786910](https://pubmed.ncbi.nlm.nih.gov/11786910/) |
| Cytokine IL-6 Treatment | Centrally acting IL-6 exerts anti-obesity effects in rodents. | [11786910](https://pubmed.ncbi.nlm.nih.gov/11786910/); |
| IL-6 Neutralizing  Antibody Treatment | IL-6 may indirectly cause muscle wasting. | [20871233](https://pubmed.ncbi.nlm.nih.gov/20871233/) |
| IL-6 KO under  Tumor-bearing | Centrally acting IL-6 exerts anti-obesity effects in rodents; Apc^Min/+^/IL-6^-/-^ mice did not lose gastrocnemius muscle mass or epididymal fat pad mass while overall polyp number decreased compared with Apc^Min/+^ mice. | [11786910](https://pubmed.ncbi.nlm.nih.gov/11786910/)  [18056981](https://pubmed.ncbi.nlm.nih.gov/18056981/) |
| IL-6 Overexpression  under Tumor-bearing | Administration of an IL-6 receptor antibody to cachectic male Apc^Min/+^ mice can attenuate further cachexia progression by increasing body weight. IL-6 over-expression in pre-cachectic mice accelerated body weight loss and muscle wasting. IL-6 overexpression did not induce cachexia in non-tumor-bearing mice. | [25555992](https://pubmed.ncbi.nlm.nih.gov/25555992/);  [22769563](https://pubmed.ncbi.nlm.nih.gov/22769563/);  [18056981](https://pubmed.ncbi.nlm.nih.gov/18056981/) |
