# Supplementary material for: MMP12 knockout prevents weight and muscle loss in tumor-bearing mice
Source: BMC Cancer. 2021 Dec 4;21:1297. doi: 10.1186/s12885-021-09004-y (PMC8642861; doi:10.1186/s12885-021-09004-y)
Supplement: Supplementary file 2 — Additional file 2. [file 12885_2021_9004_MOESM2_ESM.docx]

1. (Figure 4B, C) RAW264.7 cells co-cultured with MC38 cells for 72 hours.

Representative western blots showing the secreted MMP12 protein levels from RAW264.7 cell lines (1-2×10^5^) cultured alone or co-cultured with MC38 cell lines (control, 1x10^4^, 3x10^4^, 5x10^4^). β-Actin as the internal control.







β-Actin-43KD MMP12-54KD

1. (Figure 4D, E) RAW264.7 cells co-cultured with CT26 cells for 72 hours

Representative western blots showing the secreted MMP12 protein levels from RAW264.7 cell lines (1-2×10^5^) cultured alone or co-cultured with CT26 cell lines (control, 1x10^4^, 3x10^4^, 5x10^4^).







GAPDH-37KD MMP12-54KD

1. (Figure 4G, H) IL-6 Treatment of Macrophages RAW264.7 cells

RAW264.7 cells were seeded into 6-well plates and treated with increasing doses of IL-6 (0, 2, 5, 10, 30 ng/ mL) for 72h. RAW264.7 cells were seeded in 6-well plates and treated with increasing doses of IL-6 (0, 2, 5, 10, 30 ng/ mL) for 72 hours. Cells incubated with fresh media were used as the untreated negative controls ([Figure 4F](#FFFF)).







GAPDH -37KD MMP12-54KD
